# Supplementary material for: Paracrine cellular senescence exacerbates biliary injury and impairs regeneration
Source: Nat Commun. 2018 Mar 9;9:1020. doi: 10.1038/s41467-018-03299-5 (PMC5844882; doi:10.1038/s41467-018-03299-5)
Supplement: Supplementary file 1 — Supplementary Information [file 41467_2018_3299_MOESM1_ESM.pdf]

## SUPPLEMENTARY FIGURES

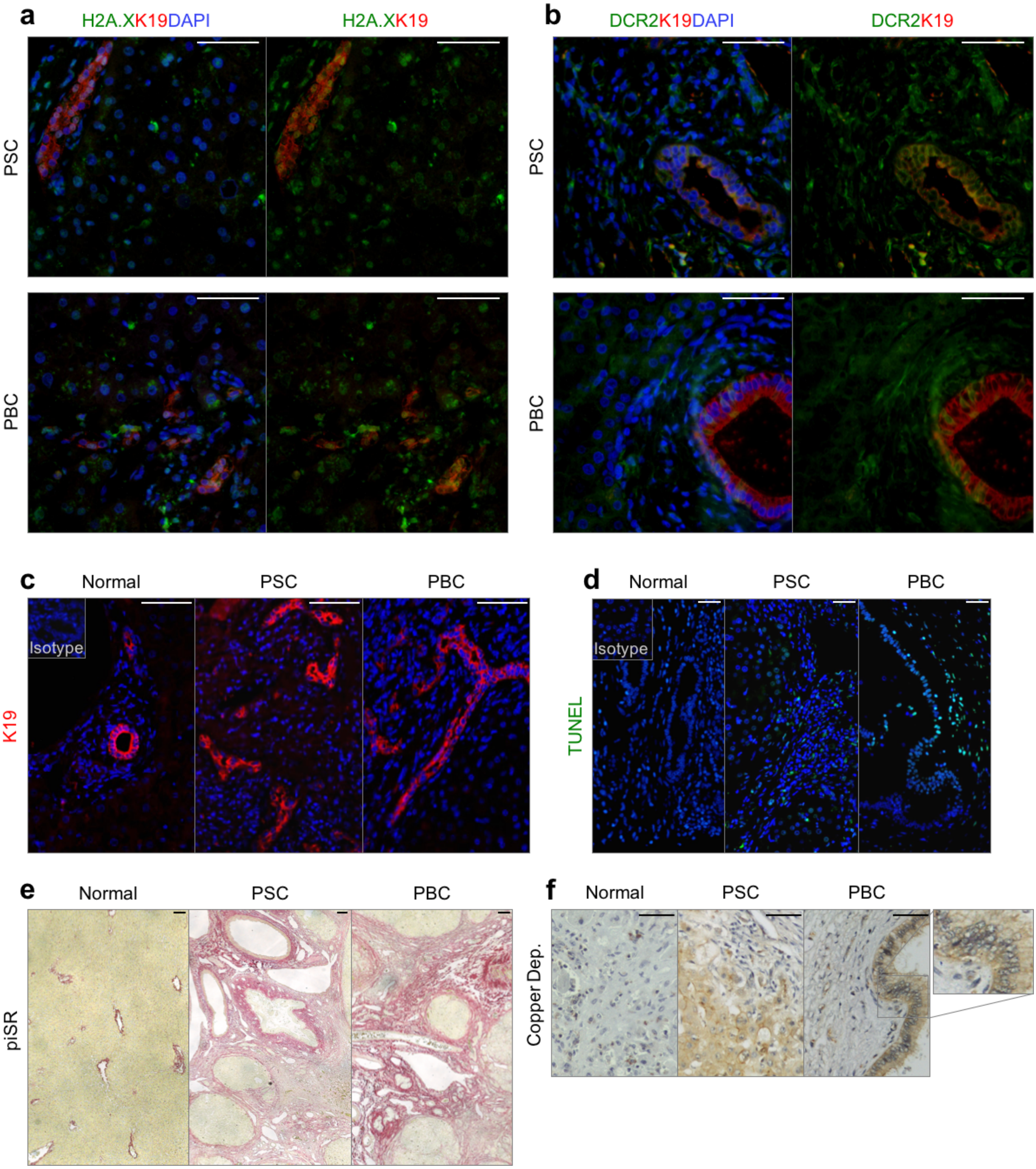

**Supplementary Figure 1. Characteristics of PSC/PBC**

Explanted human livers diagnosed as Normal, PSC and PBC respectively (series of N=7 per group).

**(a)** H2A.X (green) is present in K19-cholangiocytes (red) and surrounding hepatocytes in PSC and PBC. Right, same images without DAPI. **(b)** DCR2 (green) is present in K19-cholangiocytes (red) and surrounding hepatocytes in PSC and PBC. Right, same images without DAPI. **(c)** Keratin19-positive cholangiocytes (red) are increased in PSC/PBC, in a ductular reaction pattern. **(d)** Representative areas of TUNEL staining (green) in bile ducts of PSC/PBC compared with normal explants. **(e)** Increased deposition of collagen in PSC/PBC assessed by PicroSirius Red staining. **(f)** Copper deposition in bile ducts and hepatic parenchyma in PSC and PBC.

Scale bars = 50µm

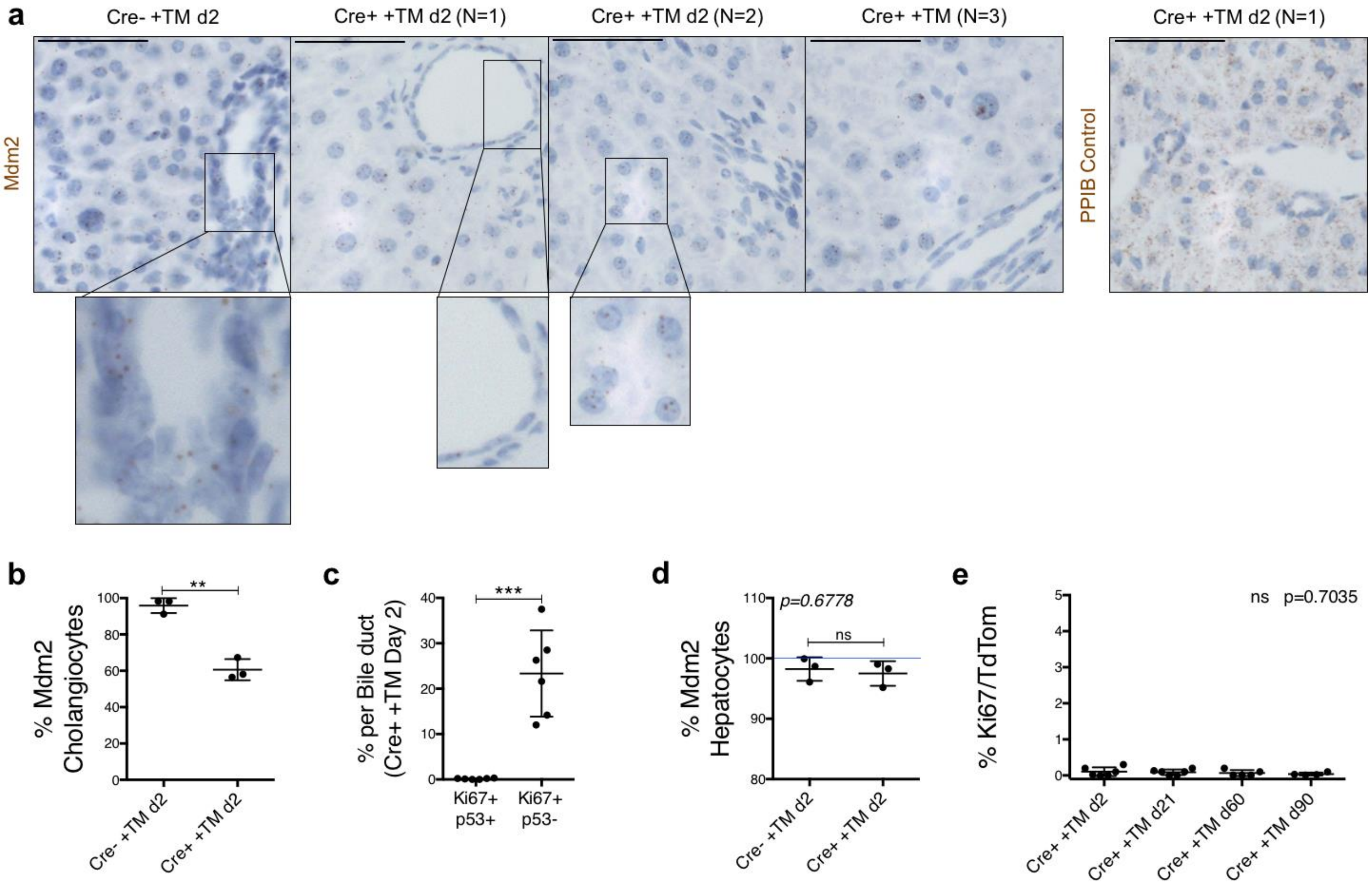
a
Cre- +TM d2
Cre+ +TM d2 (N=1)
Cre+ +TM d2 (N=2)
Cre+ +TM (N=3)
Cre+ +TM d2 (N=1)
Mdm2
PPIB Control
b
% Mdm2 Cholangiocytes
**
Cre- +TM d2
Cre+ +TM d2
c
% per Bile duct (Cre+ +TM Day 2)
***
Ki67+ p53+
Ki67+ p53-
d
% Mdm2 Hepatocytes
p=0.6778
ns
Cre- +TM d2
Cre+ +TM d2
e
% Ki67/TdTom
ns p=0.7035
Cre+ +TM d2
Cre+ +TM d21
Cre+ +TM d60
Cre+ +TM d90

**Supplementary Figure 2. Characterisation of senescence in K19-Mdm2$^{flox/flox}$tdTom$^{LSL}$ mice**

**(a)** ISH by RNAscope for *Mdm2* in Cre- +TM (N=3) and Cre+ +TM (N=3) at day 2. For the Cre- +TM, the selected area shows the presence of *Mdm2* in cholangiocytes. For the Cre+ +TM, representative images of the three mice (N=1, 2 and 3) show the loss of Mdm2 in cholangiocytes (in N=1) while hepatocytes still maintain *Mdm2* expression (as seen in the magnified area of N=2). Right *PPIB* positive control (N=1). Scale bars = 50µm. **(b)** Quantification of *Mdm2* in cholangiocytes (in the *Mdm2*-RNAscope images) shows the loss of *Mdm2* after the induction of the model. ** denotes $p$ <0.01, (Mean ± SEM) Student's t-test, (N=3 per group) **(c)** Quantification of the total percentage of p53-positive and p53-negative cholangiocytes that proliferate in the Cre+ +TM mice at day 2. This result shows that p53-positive cells do not proliferate while p53-negative cells do. *** denotes $p$ <0.001, (Mean ± SEM) Student's t-test, (N=6 per group). **(d)** Quantification of *Mdm2* in hepatocytes (in the *Mdm2*-RNAscope images) shows that hepatocytes maintain similar levels of *Mdm2* after the induction of the model. Student's t-test. (N=3 per group). The blue line depicts the level of *Mdm2* for both groups. **(e)** Quantification of proliferation (assessed by Ki67) in TdTom-positive cholangiocytes shows no significant differences over the course of 90 days. ANOVA, Sidak's multiple comparisons test. N=4-6 per group.

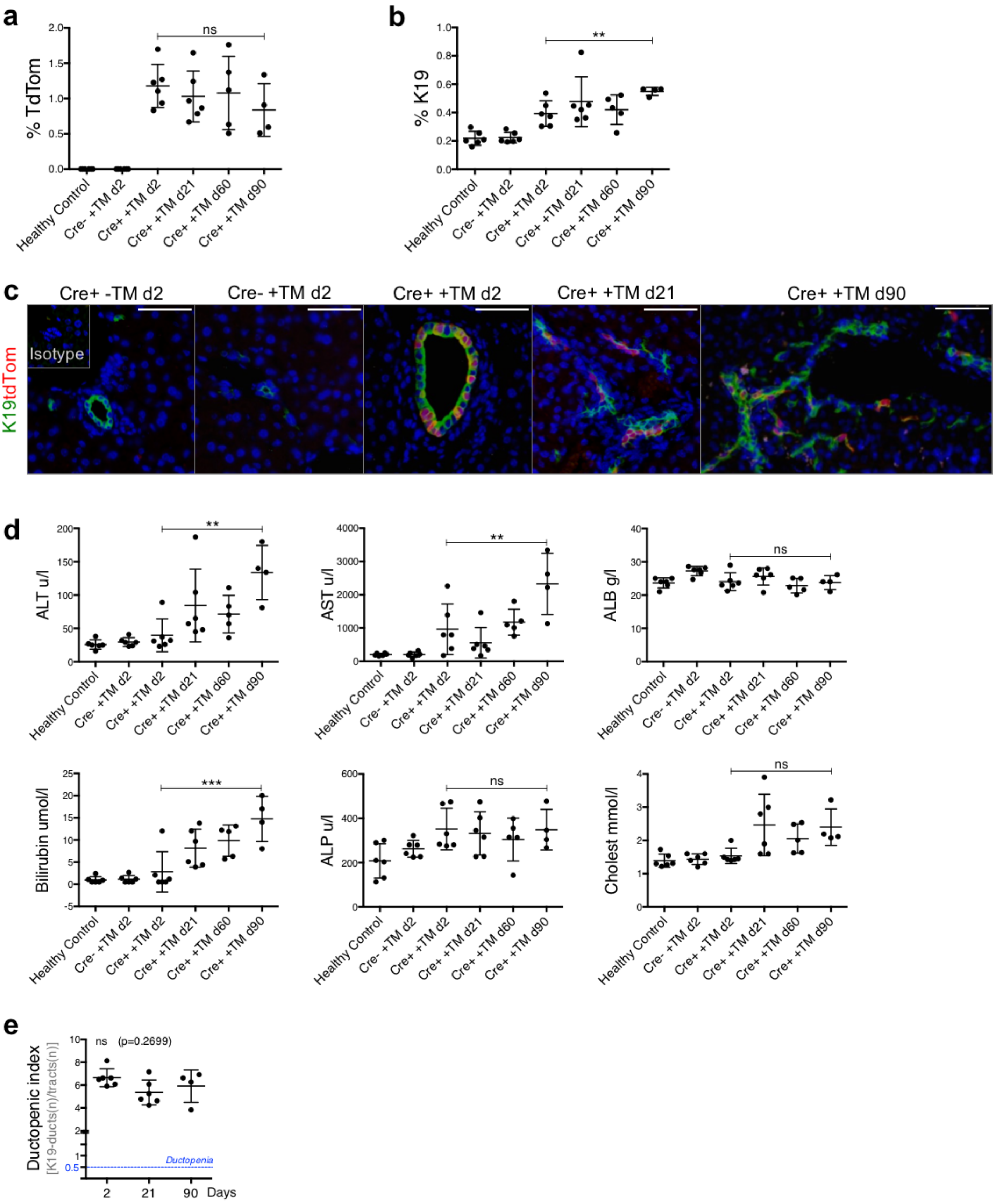

a
% TdTom
ns
Healthy Control
Cre- +TM d2
Cre+ +TM d2
Cre+ +TM d21
Cre+ +TM d60
Cre+ +TM d90
b
% K19
**
c
Cre+ -TM d2
Cre- +TM d2
Cre+ +TM d2
Cre+ +TM d21
Cre+ +TM d90
K19tdTom
Isotype
d
ALT u/l
AST u/l
ALB g/l
Bilirubin umol/l
ALP u/l
Cholest mmol/l
***
e
Ductopenic index
[K19-ducts(n)/tracts(n)]
ns (p=0.2699)
Ductopenia
Days

**Supplementary Figure 3. Characterisation of senescence in K19-Mdm2$^{flox/flox}$tdTom$^{LSL}$ mice**

Long term assessment of the senescent response.

**(a)** Quantification of the total percentage of TdTom-positive cells increases after induction of the model at day 2 but shows no significant differences over the course of 90 days. ANOVA, Sidak's multiple comparisons test. **(b)** Quantification of the total percentage of K19 significantly increases over the course of 90 days. ** denotes *p* <0.01, (Mean ± SEM). ANOVA, Sidak's multiple comparisons test. N=4-6 per group. **(c)** Increase of the K19-population (green) over the course of 90 days. Notice the ductular reaction pattern surrounding the TdTom-positive cholangiocytes (red) at day 90. N=4-6 per group. Scale bars = 50µm. **(d)** Liver transaminases in healthy control, Cre- +TM at day 2 and Cre+ +TM at day 2, 21, 60 and 90 after tamoxifen administration. N=4-6 per group. **(e)** Ductopenic index shows no significant alterations in the course of 90 days. The blue line depicts the baseline level of ductopenia. ANOVA, Sidak's multiple comparisons test. (N=4-6 per group).

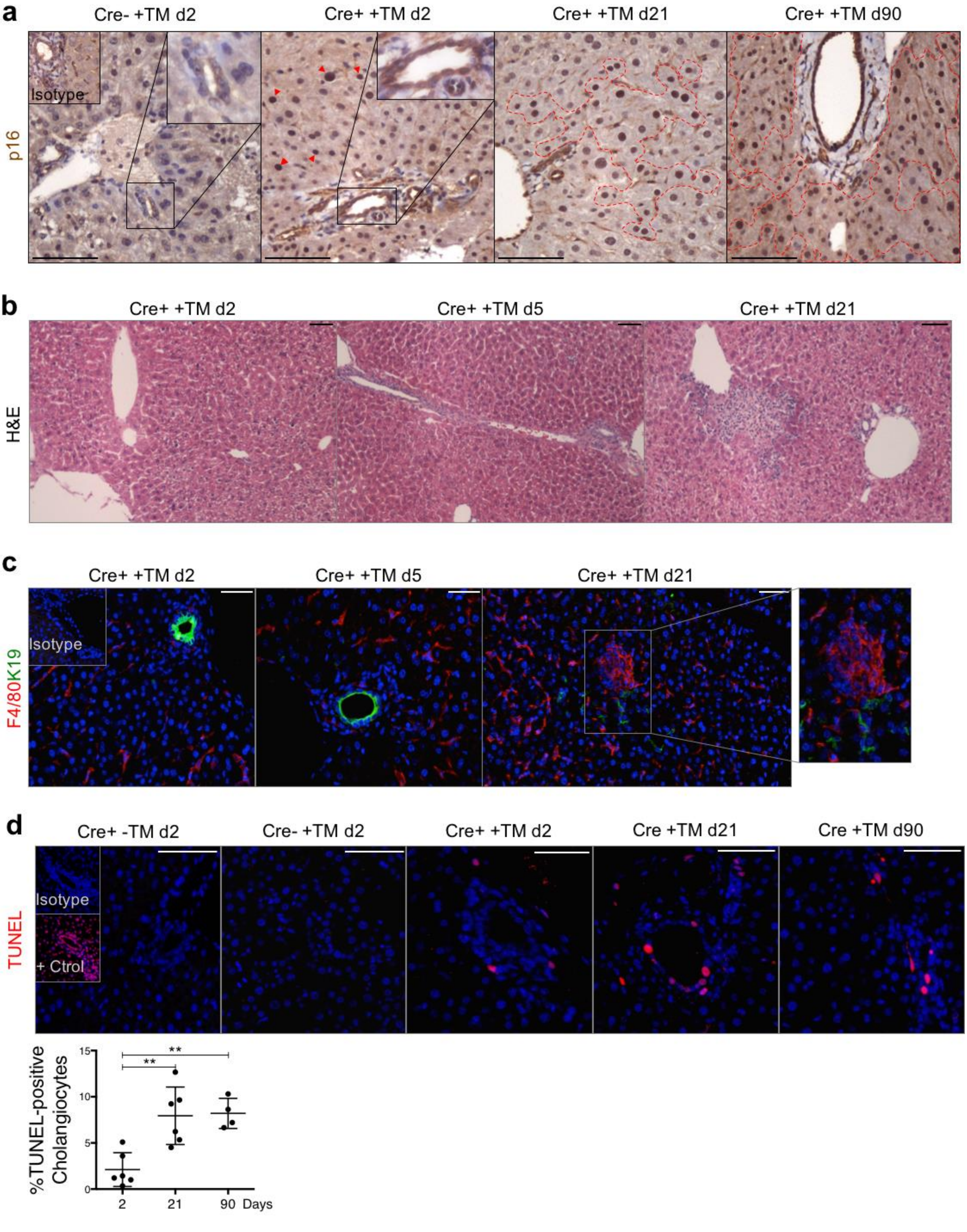
a
Cre- +TM d2
Cre+ +TM d2
Cre+ +TM d21
Cre+ +TM d90
p16
Isotype
b
Cre+ +TM d2
Cre+ +TM d5
Cre+ +TM d21
H&E
c
Cre+ +TM d2
Cre+ +TM d5
Cre+ +TM d21
F4/80K19
Isotype
d
Cre+ -TM d2
Cre- +TM d2
Cre+ +TM d2
Cre +TM d21
Cre +TM d90
TUNEL
Isotype
+ Ctrol
%TUNEL-positive Cholangiocytes
**
**
15
10
5
0
2
21
90
Days

**Supplementary Figure 4. Characterisation of senescence in K19-Mdm2$^{flox/flox}$tdTom$^{LSL}$ mice**

**(a)** p16 immunohistochemistry shows expression of p16 in cholangiocytes at day 2, 21 and 90 after induction of the model. Some hepatocytes surrounding the p16-positive cholangiocytes also present p16 expression after induction of the model (red arrows and red dotted line). **(b)** Mononuclear infiltrates in the K19-Mdm2$^{flox/flox}$tdTom$^{LSL}$ model at increasing time points. **(c)** F4/80 positive macrophages (red) are found in the proximities of K19 bile ducts (green) in the K19-Mdm2$^{flox/flox}$tdTom$^{LSL}$ model at increasing time points. **(d)** TUNEL staining (red) shows an increase in apoptotic cholangiocyte nuclei in the Cre+ mice 21 days after induction. N= 4-6 per group. Quantification of TUNEL-positive cholangiocytes over the course of 90 days after induction of the model. The image includes isotype control and DNAseI-positive control. ** denotes $p$ <0.01, (Mean ± SEM). ANOVA, Sidak's multiple comparisons test.

N= 4-6 per group. Scale bars = 50µm.

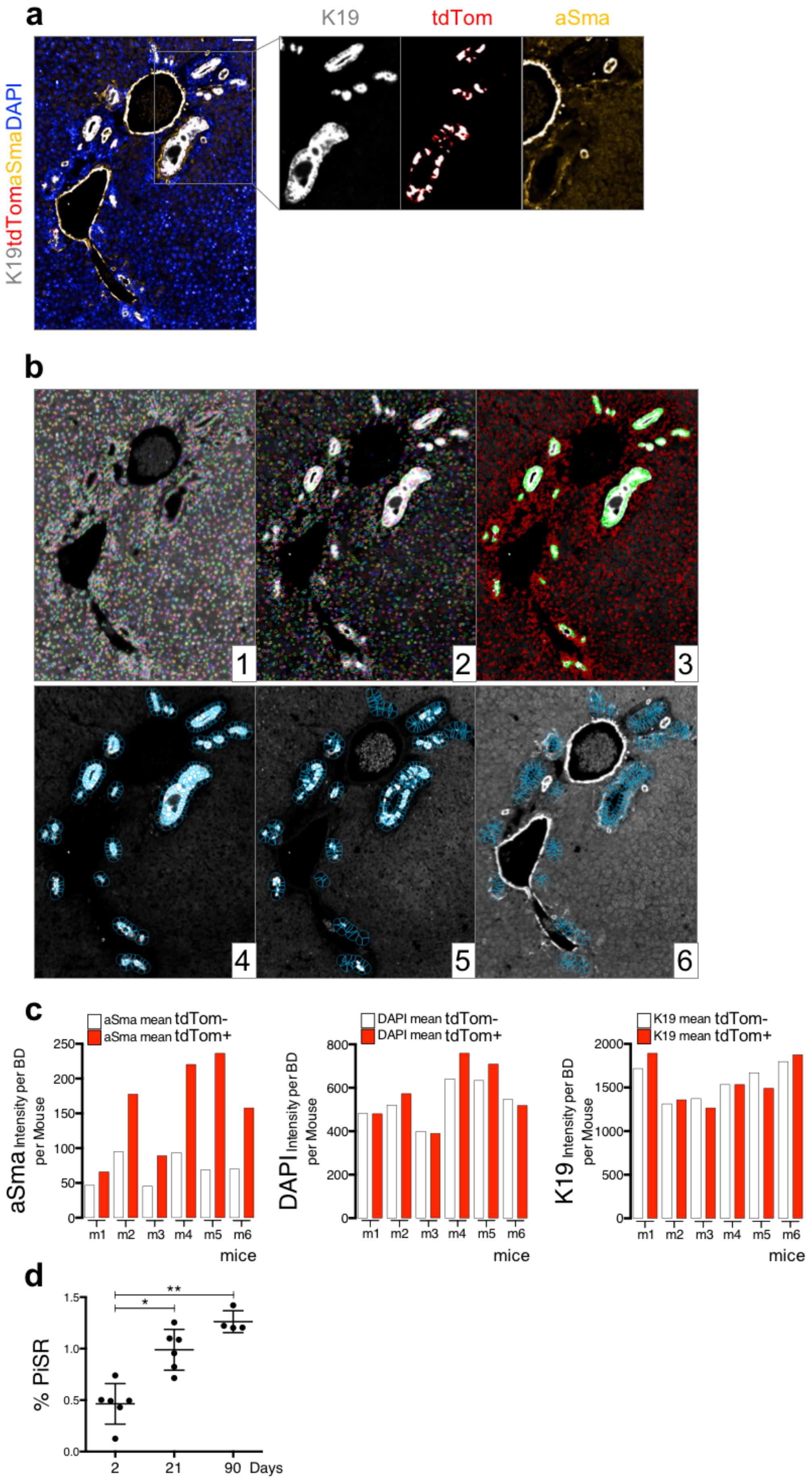

a
K19tdTomaSmaDAPI
K19
tdTom
aSma
b
1
2
3
4
5
6
c
aSma mean tdTom-
aSma mean tdTom+
aSma Intensity per BD per Mouse
DAPI mean tdTom-
DAPI mean tdTom+
DAPI Intensity per BD per Mouse
K19 mean tdTom-
K19 mean tdTom+
K19 Intensity per BD per Mouse
m1 m2 m3 m4 m5 m6
mice
d
% PiSR
2 21 90 Days

**Supplementary Figure 5 Recruitment of αSMA-positive cells in the proximities of TdTom-positive senescent cholangiocytes**

**(a)** Triple immunostaining of K19-cholangiocytes (grey), TdTom-positive senescent cells (red), αSMA-positive cells (yellow) and DAPI-positive nuclei (blue) at day 21 post-induction. **(b)** Example of the Columbus sequential analysis for the triple staining: Find nuclei (DAPI staining); Measure K19 in nuclei population; Select K19 positive nuclei; Select area around K19 positive nuclei region (plus 15 µm); Measure TdTom intensity in the 15 µm K19-positive nuclei region; Measure αSma intensity in the 15 µm K19-positive nuclei region. **(c)** Internal controls for the Columbus Analysis. From left to right:

- αSMA intensity in proximity to RFP-positive vs RFP-negative cells per mouse show differences between the RFP-positive (senescent cholangiocytes) and the RFP-negative (normal cholangiocytes). Each couple of white and red bars represent one mouse.

- Similar ratio of DAPI-nuclei in RFP-positive and RFP-negative cells shows that the number of nuclei counted per bile duct are similar for both groups, reducing the false positive ratio.

- Similar ratio of K19 intensity in RFP-positive and RFP-negative cells shows that the intensity of K19-postiive nuclei per bile duct is similar for both groups, reducing the false positive ratio.

m1, m2 (up to m6) represents one mouse. Total mice N=6. No statistical analysis is performed in this set of samples.

**(d)** Quantification of collagen deposition assessed by PiSR staining over the course of 90 days after the induction of the model. * denotes $p$ <0.05, ** denotes $p$ <0.01, (Mean ± SEM). ANOVA, Sidak's multiple comparisons test. N=4-6 per group.

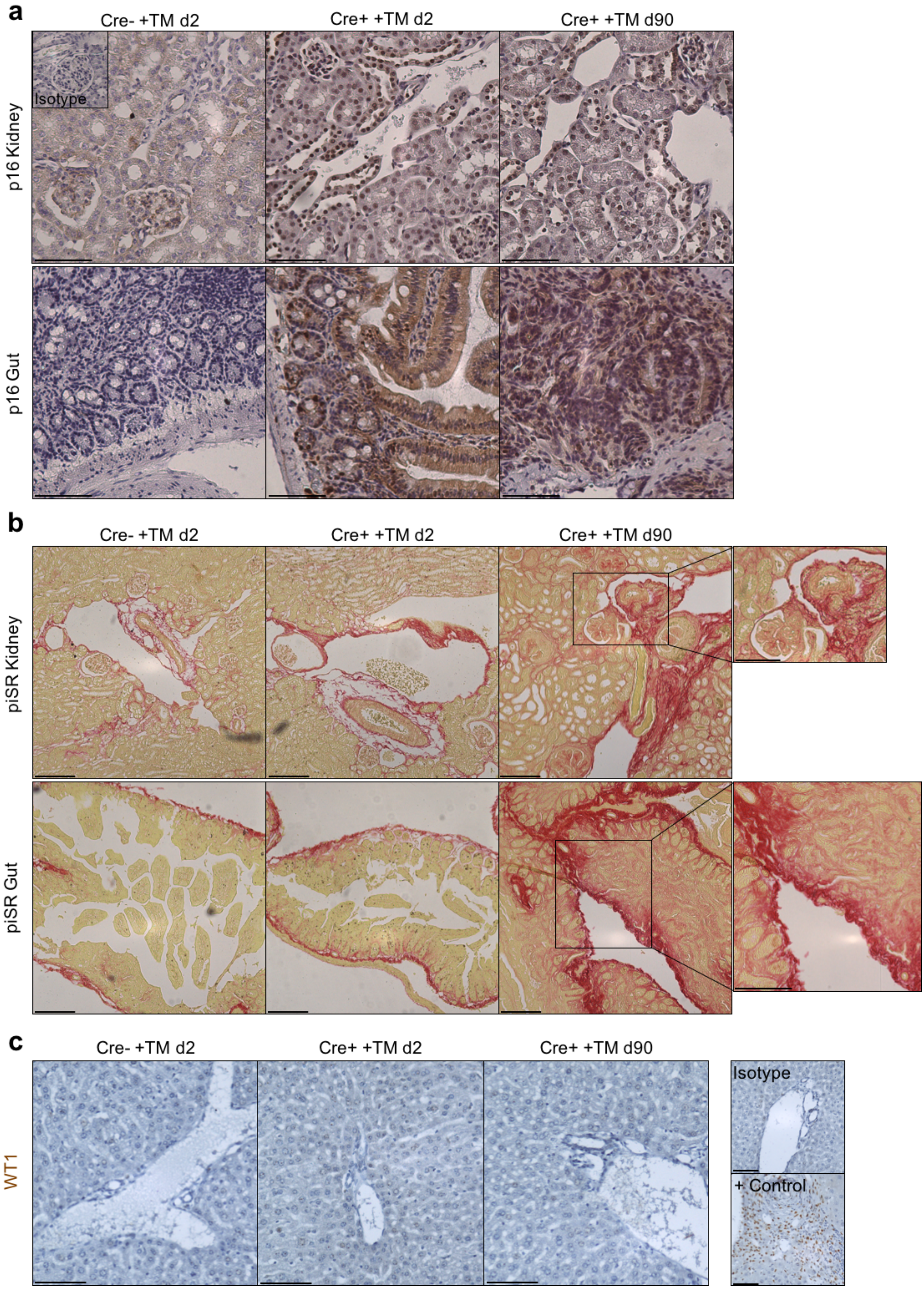
a
Cre- +TM d2
Cre+ +TM d2
Cre+ +TM d90
Isotype
p16 Kidney
p16 Gut
b
Cre- +TM d2
Cre+ +TM d2
Cre+ +TM d90
piSR Kidney
piSR Gut
c
Cre- +TM d2
Cre+ +TM d2
Cre+ +TM d90
WT1
Isotype
+ Control

**Supplementary Figure 6. Characterisation of senescence in K19-Mdm2$^{flox/flox}$tdTom$^{LSL}$ mice**

From left to right:

Left: Cre-negative mice at day 2 after tamoxifen administration.

Center: Cre-positive mice at day 2 after tamoxifen administration

Right: Cre-positive mice at day 90 after tamoxifen administration.

**(a)** p16 immunohistochemistry in Cre- +TM at day 2 shows low expression of p16 in kidney and gut epithelia. After induction, p16 is expressed in both epithelia at day 2 and day 90. **(b)** Collagen accumulation assessed by PiSR staining shows increased deposition over the course of time in gut and kidney epithelia. **(c)** Immunohistochemistry for Wilms Tumor 1 (WT1) shows no involvement of mesenchymal cells in our model. This image includes isotype control and a positive control (Glisson Capsule of K19-Mdm2$^{flox/flox}$tdTom$^{LSL}$ mice, connective tissue septa). Scale bars = 50µm. (N=4-6 per group).

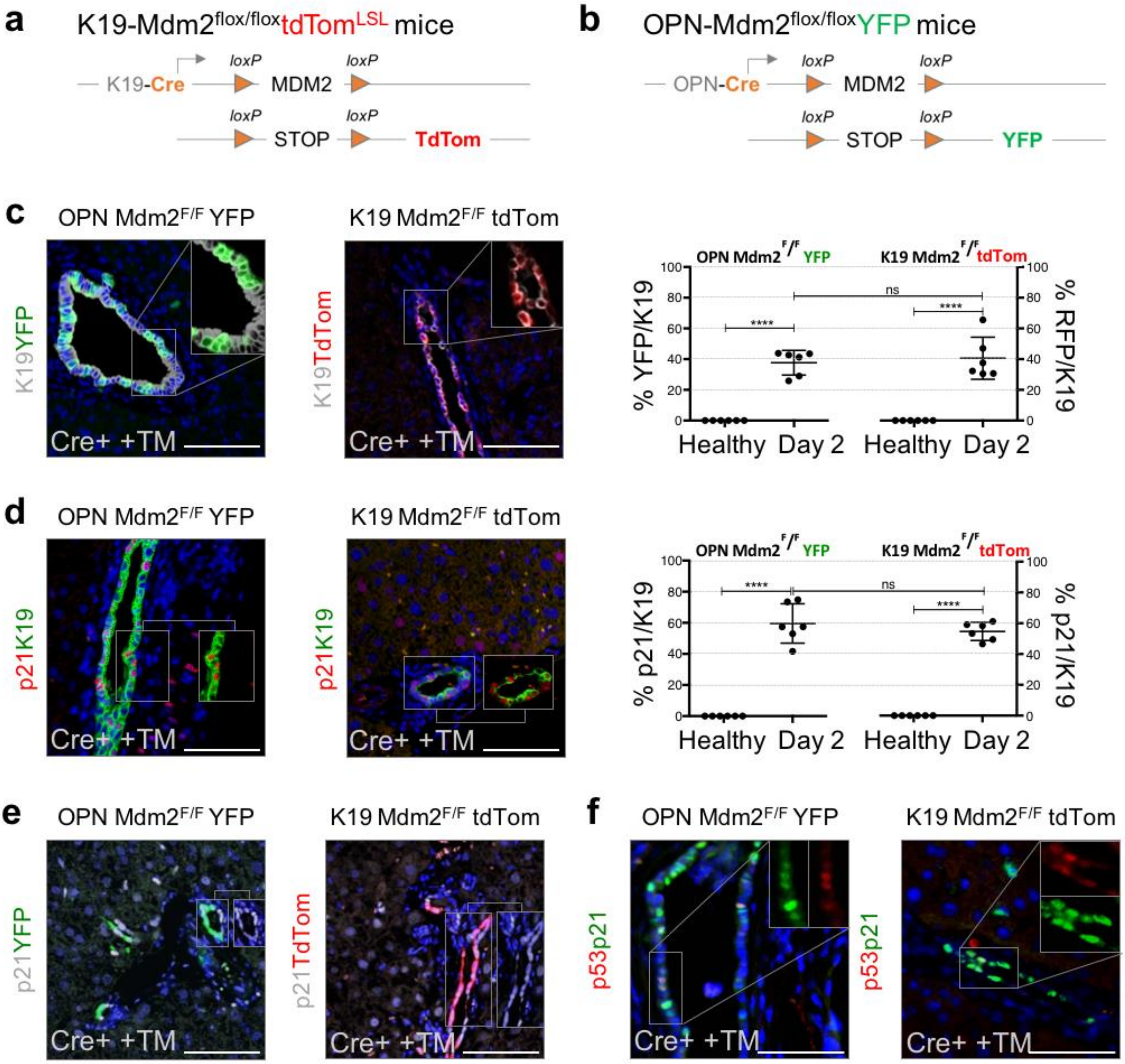
a
K19-Mdm2flox/floxtdTomLSL mice
K19-Cre
loxP
MDM2
loxP
loxP
STOP
loxP
TdTom
b
OPN-Mdm2flox/floxYFP mice
OPN-Cre
loxP
MDM2
loxP
loxP
STOP
loxP
YFP
c
OPN Mdm2F/F YFP
K19 Mdm2F/F tdTom
K19YFP
K19TdTom
Cre+ +TM
OPN Mdm2F/F YFP
K19 Mdm2F/F tdTom
% YFP/K19
% RFP/K19
ns
****
Healthy
Day 2
d
OPN Mdm2F/F YFP
K19 Mdm2F/F tdTom
p21K19
Cre+ +TM
% p21/K19
ns
****
Healthy
Day 2
e
OPN Mdm2F/F YFP
K19 Mdm2F/F tdTom
p21YFP
p21TdTom
Cre+ +TM
f
OPN Mdm2F/F YFP
K19 Mdm2F/F tdTom
p53p21
Cre+ +TM

**Supplementary Figure 7. Comparison between K19-Mdm2$^{flox/flox}$tdTom$^{LSL}$ and OPN-Mdm2$^{flox/flox}$YFP mouse models show similar senescent response**

**(a)** Schematic representation of K19-Mdm2$^{flox/flox}$tdTom$^{LSL}$ model. **(b)** Schematic representation of OPN-Mdm2$^{flox/flox}$YFP model. **(c)** Primary senescence in cholangiocytes is similar in both models. Images show YFP-positive cholangiocytes (green) and tdTom-positive (red) K19-positive cholangiocytes (grey). Upper corner of the image shows a digital magnification of representative areas. Far right, quantification of the number of YFP per K19-positive cholangiocytes (OPN-Mdm2$^{flox/flox}$YFP model) vs tdTom per K19-positive cholangiocytes (K19-Mdm2$^{flox/flox}$tdTom$^{LSL}$). Images show uninduced (Cre+ -TM) *versus* induced (Cre+ +TM) mice at day 2 after last tamoxifen injection. **(d)** Image of p21-positive (red) cholangiocytes (green) in both models. Far right, quantification. **(e)** Representative images of p21 (grey) paracrine senescence in both models at day 2 after induction. Note that while YFP (green) / tdTom (red) expression is restricted to the bile ducts, p21 is also expressed in the surrounding cells. **(f)** p53-positive (red) p21-positive (green) cholangiocytes show paracrine response in cholangiocytes for both models at day 2 after induction.

**** denotes $p$ <0.0001, (Mean ± SEM). ANOVA, Sidak's multiple comparisons test. (N=6 per group).

Scale bars = 50µm.

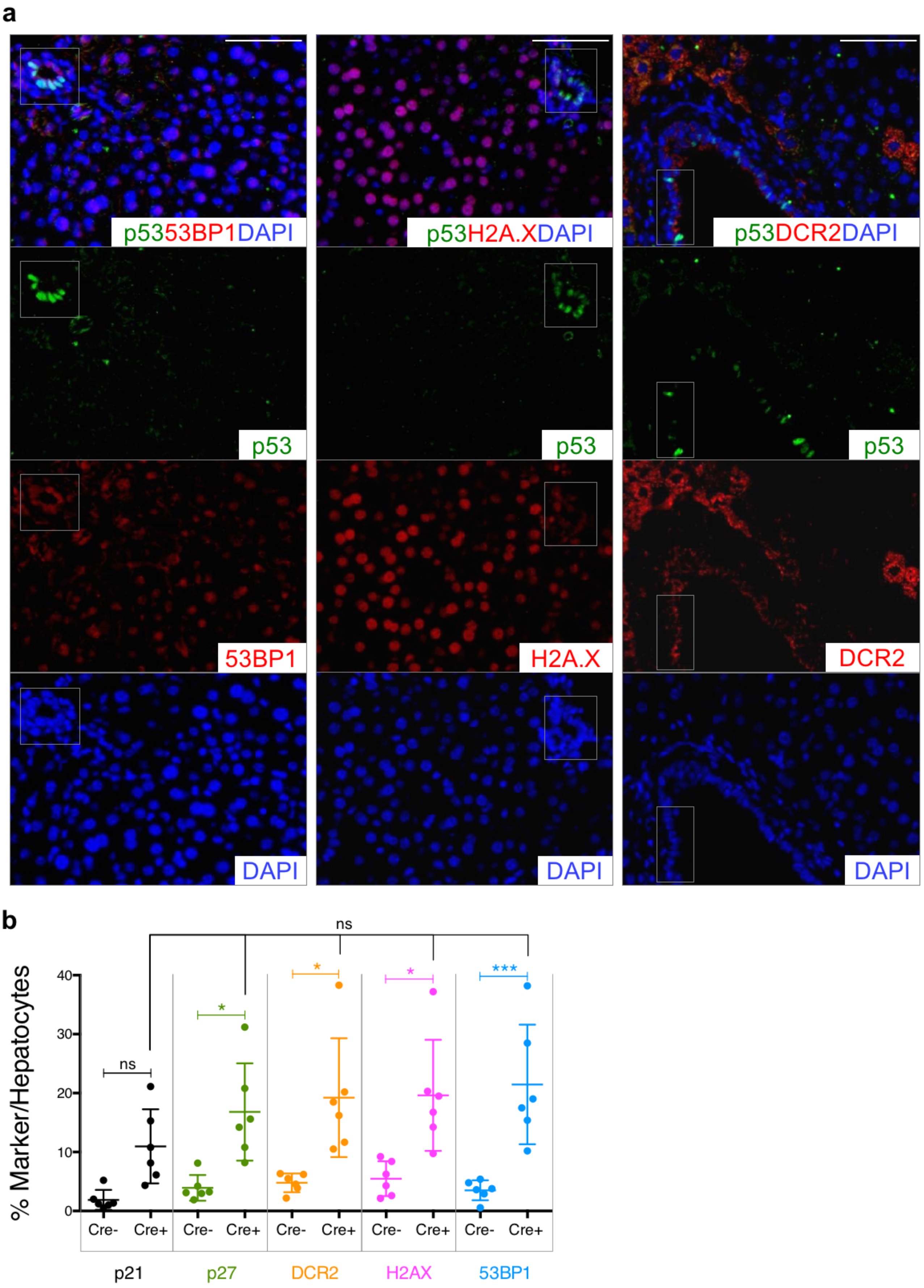

a
p5353BP1DAPI
p53H2A.XDAPI
p53DCR2DAPI
p53
p53
p53
53BP1
H2A.X
DCR2
DAPI
DAPI
DAPI
b
% Marker/Hepatocytes
ns
ns
*
*
*
***
0
10
20
30
40
Cre-
Cre+
Cre-
Cre+
Cre-
Cre+
Cre-
Cre+
Cre-
Cre+
p21
p27
DCR2
H2AX
53BP1

**Supplementary Figure 8. Senescent markers in the hepatocytes of the induced K19 K19-Mdm2$^{flox/flox}$tdTom$^{LSL}$ model**

**(a)** Single channels for Figure 3e (Fig 3. Paracrine senescence in the model is TGFβ-dependent). Double immunostaining for p53 (green) and several senescent markers in Cre+ +TM mice at day 2 after induction (N=6 per group). From left to right: 53BP1, H2A.X and DCR2 (red). From up-down: Merge, p53, senescent marker, DAPI. Scale bars = 50µm. **(b)** Quantification of several senescent markers in the immunofluorescences of Cre- +TM vs Cre+ +TM, 2 days after tamoxifen administration. From left to right: p21, p27, DCR2, H2A.X and 53BP1. * denotes $p$ <0.05, (Mean ± SEM) Student's t-test, (N=6 per group)

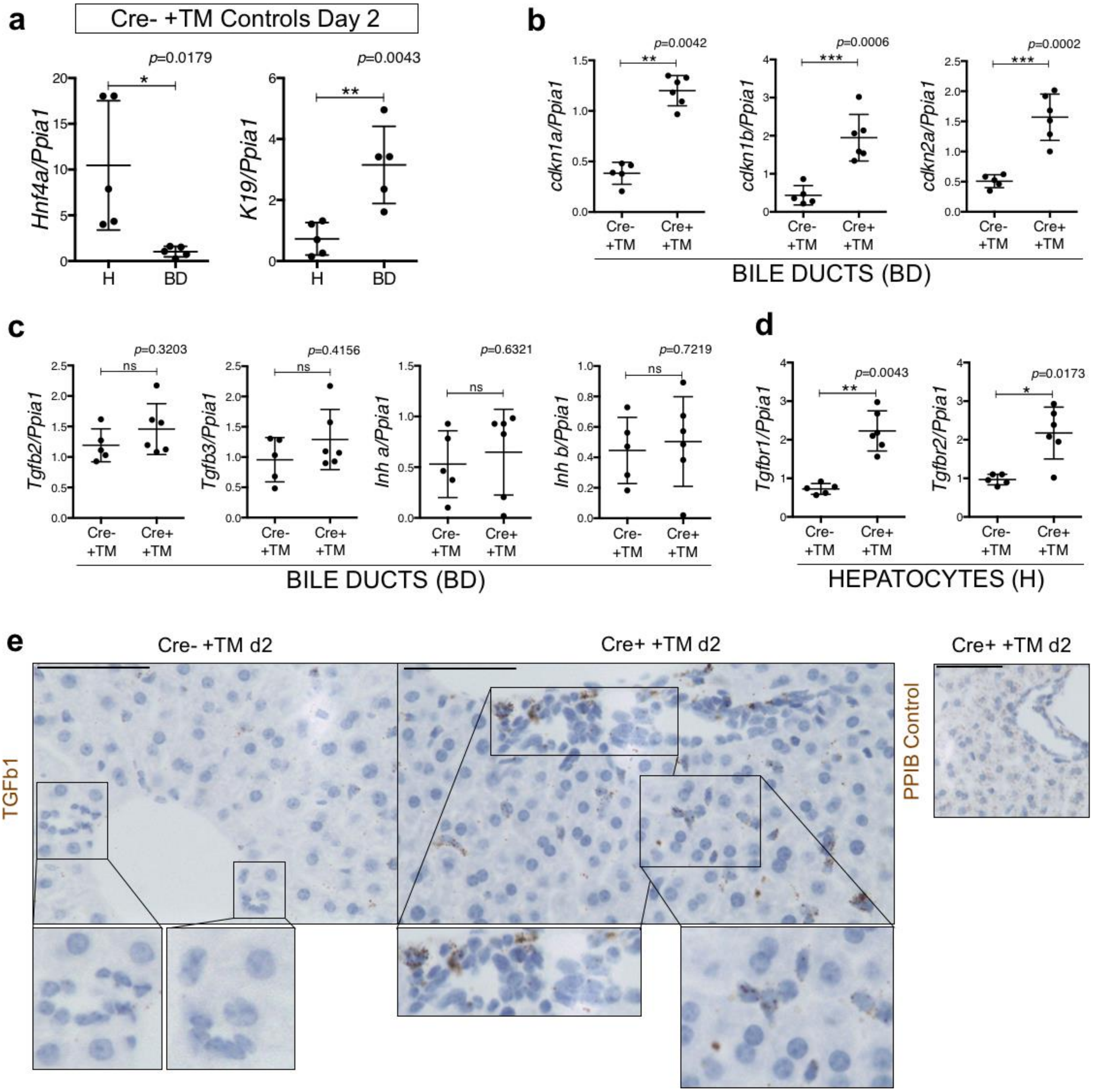
a
Cre- +TM Controls Day 2
p=0.0179
p=0.0043
Hnf4a/Ppia1
K19/Ppia1
H
BD
b
p=0.0042
p=0.0006
p=0.0002
cdkn1a/Ppia1
cdkn1b/Ppia1
cdkn2a/Ppia1
Cre- +TM
Cre+ +TM
BILE DUCTS (BD)
c
p=0.3203
p=0.4156
p=0.6321
p=0.7219
ns
Tgfb2/Ppia1
Tgfb3/Ppia1
Inh a/Ppia1
Inh b/Ppia1
BILE DUCTS (BD)
d
p=0.0043
p=0.0173
Tgfbr1/Ppia1
Tgfbr2/Ppia1
HEPATOCYTES (H)
e
Cre- +TM d2
Cre+ +TM d2
Cre+ +TM d2
TGFb1
PPIB Control

**Supplementary Figure 9. Paracrine senescence effects in the K19-Mdm2$^{flox/flox}$tdTom$^{LSL}$ model**

**(a)** qRT-PCR analysis of *HNF4a* and *K19* in the isolated bile ducts (N=5) and hepatocytes (N=5) of Cre- +TM and Cre+ +TM mice day 2 after induction. Hepatocytes have high expression of the hepatocyte marker *HNF4a* but low expression of the epithelial marker *K19*. On the other hand, Bile ducts have low expression of *HNF4a* and high expression of *K19*. * denotes $p$ <0.05, ** denotes $p$ <0.01 (Mean± SEM). Mann-Whitney test. **(b)** qRT-PCR of cell-cycle related genes *cdkn1a* (p21), *cdkn1b* (p27) and *cdkn2a* (p16) show a significant increase in isolated bile ducts of Cre+ +TM (N=6) group, when compared with the Cre- +TM group (N=5) (day 2 after induction). ** denotes $p$ <0.01, *** denotes $p$ <0.001 (Mean± SEM). Mann-Whitney test. **(c)** qRT- PCR of other members of the TGFb signaling pathway, such as *Tgfb2*, *Tgfb3*, *Inha* (Inhibin a) and *Inhb* (Inhibin b) show no significant differences in isolated bile ducts of Cre+ +TM (N=6) group, when compared with the Cre- +TM group (N=5) (day 2 after induction). Mann-Whitney test. **(d)** qRT-PCR of *Tgfbr1* and *Tgfbr2* in isolated hepatocytes of Cre- +TM (N=5) and Cre+ +TM (N=6) mice at day 2 after induction are significantly increased. * denotes $p$ <0.05, ** denotes $p$ <0.01 (Mean± SEM). Mann-Whitney test. **(e)** ISH by RNAscope for *Tgfb1* in Cre- +TM and Cre+ +TM at day 2 (N=3 per group). For the Cre- +TM, the selected areas show the absence of *Tgfb1* in cholangiocytes. For the Cre+ +TM, selected areas are magnifications of cholangiocytes (bottom left) and hepatocytes (bottom right) that express *Tgfb1*. Notice the presence of some *Tgfb1*-positive macrophages in the Cre+ +TM group. Right *PPIB* positive control (N=1). Scale bars = 50µm

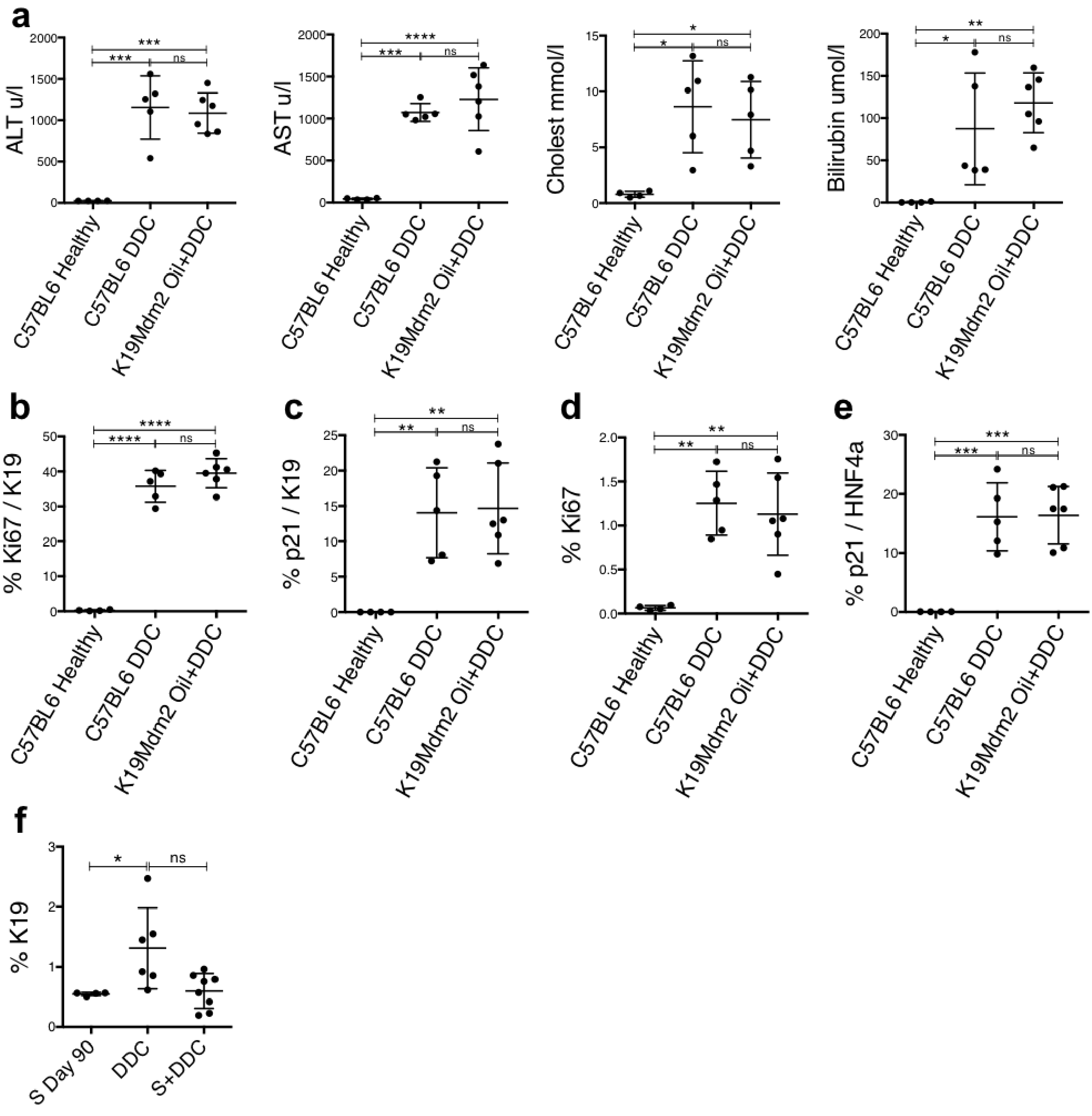
a
ALT u/l
AST u/l
Cholest mmol/l
Bilirubin umol/l
b
% Ki67 / K19
c
% p21 / K19
d
% Ki67
e
% p21 / HNF4a
C57BL6 Healthy
C57BL6 DDC
K19Mdm2 Oil+DDC
f
% K19
S Day 90
DDC
S+DDC
ns

**Supplementary Figure 10. Cellular senescence in cholangiocytes aggravates biliary injury. C57BL6 wild type controls**

This figure presents a comparison between:

- C57BL6 Healthy: wild type C57BL6 mice (the same background as the K19-Mdm2$^{flox/flox}$tdTom$^{LSL}$ model) (N=4).

- C57BL6 DDC: 1 week DDC in wild type C57BL6 mice (N=5).

- K19Mdm2 Oil+DDC: K19-Mdm2$^{flox/flox}$tdTom$^{LSL}$ model administered with the vehicle oil and maintained 1 week under DDC diet (N=6).

**(a)** Liver transaminases (alanine transaminase, aspartate aminotransferase, cholesterol and bilirubin). **(b)** Total percentage of proliferating (Ki67-positive) K19-positive cells. **(c)** Total percentage of senescent (p21-positive) K19-positive cells. **(d)** Total percentage of proliferating (Ki67-positive) cells. **(e)** Total percentage of senescent (p21-positive) HNF4a-positive hepatocytes.

**(f)** Comparison of the total percentage of K19-positive cells. From left to right: day 90 after induction of the K19-Mdm2$^{flox/flox}$tdTom$^{LSL}$ model; C57BL6 DDC (1 week DDC in wild type C57BL6 mice), and S+DDC (induced K19-Mdm2$^{flox/flox}$tdTom$^{LSL}$ maintained 1 week under DDC diet).

* denotes $p$ <0.05, ** denotes $p$ <0.01, *** denotes $p$ <0.001, *** denotes $p$ <0.001 (Mean± SEM). ANOVA, Sidak's multiple comparisons test. N=4-8 per group.

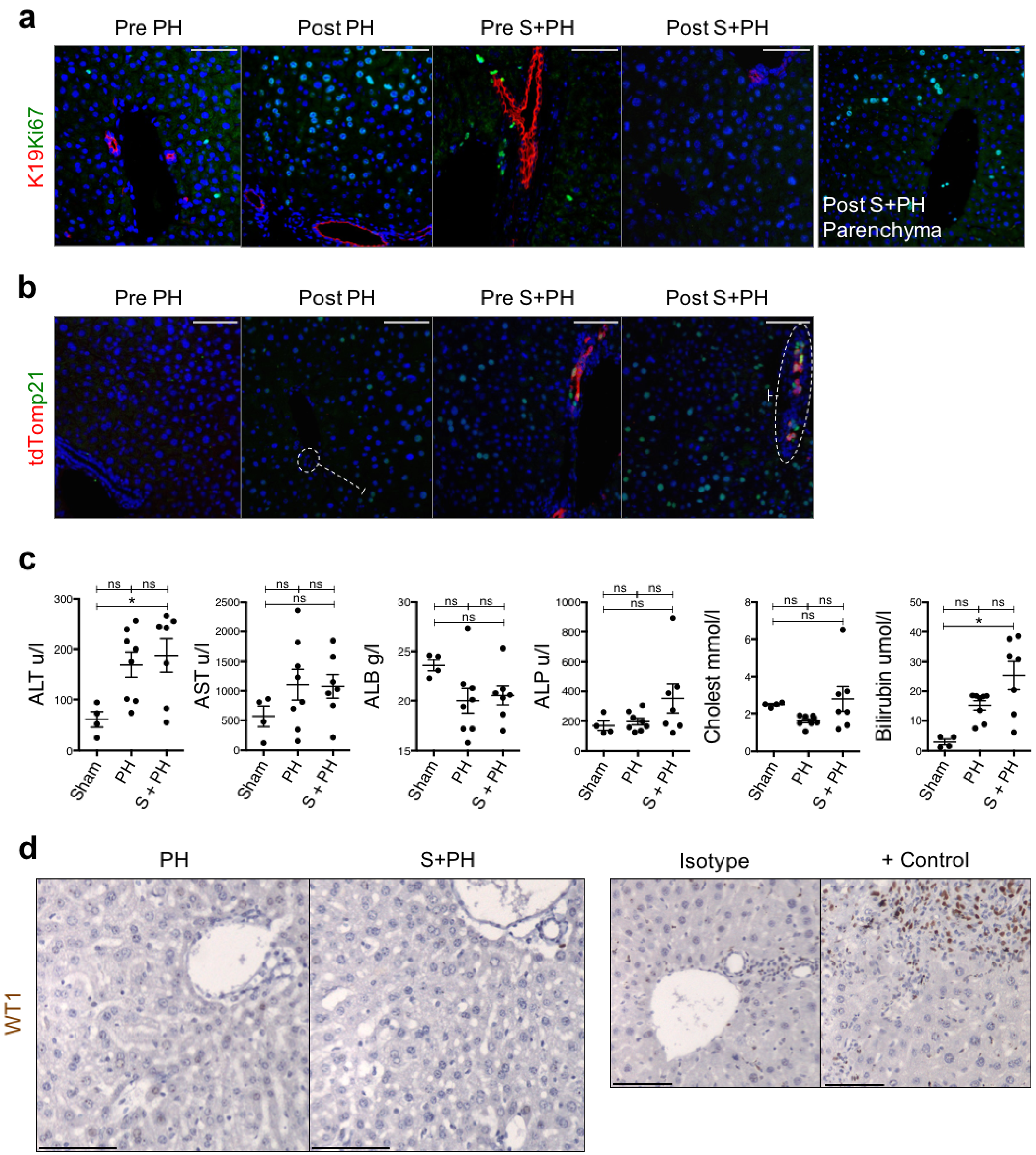
a
Pre PH
Post PH
Pre S+PH
Post S+PH
K19Ki67
Post S+PH
Parenchyma
b
Pre PH
Post PH
Pre S+PH
Post S+PH
tdTomp21
c
ALT u/l
AST u/l
ALB g/l
ALP u/l
Cholest mmol/l
Bilirubin umol/l
ns
*
Sham
PH
S + PH
d
PH
S+PH
Isotype
+ Control
WT1

**Supplementary Figure 11. Partial Hepatectomy controls**

Pre-PH group represents the resected lobes of the liver collected at the moment of the surgery. Post-PH group represents the liver at the 48h time point. N= 8 PH; 7 S+PH.

**(a)** Representative images of Ki67 (green) in K19-positive cholangiocytes (red) show decrease in the number of proliferating hepatocytes 48 hours after partial hepatectomy. **(b)** Representative images of the senescent response (p21 green, tdTom red). Notice the proximity of p21-positive cells to the bile ducts in S+PH group compared with PH group. **(c)** Liver transaminases. Notice the significant differences in Aspartate aminotransferase and bilirubin between Sham and S+PH groups. **(d)** Immunohistochemistry for Wilms Tumor 1 (WT1) shows no involvement of mesenchymal cells in our model after partial hepatectomy. This image includes isotype control and a positive control (Glisson Capsule of K19-Mdm2$^{flox/flox}$tdTom$^{LSL}$ mice, connective tissue septa).

Scale bars = 50µm

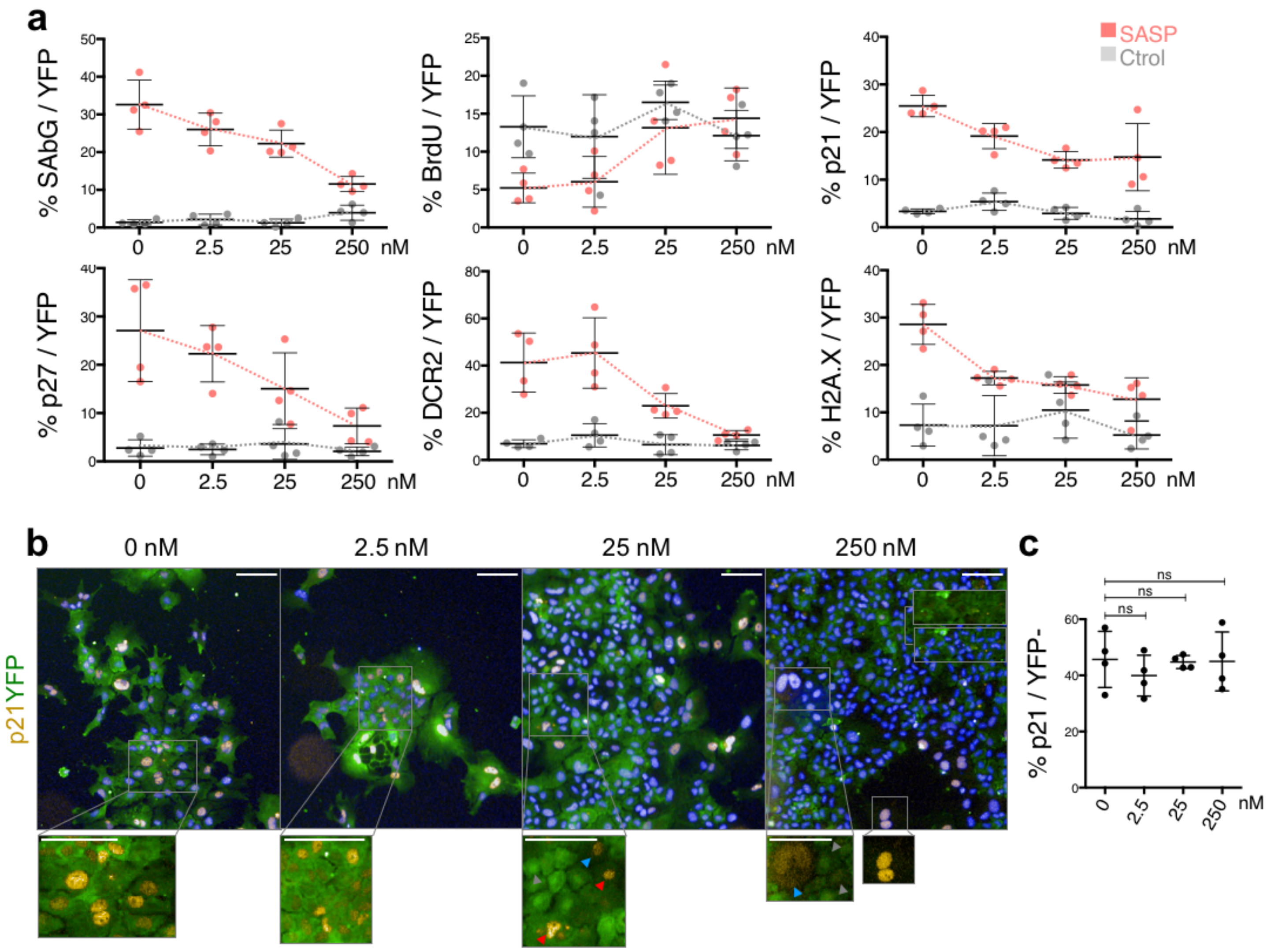
a
% SAbG / YFP
% BrdU / YFP
% p21 / YFP
% p27 / YFP
% DCR2 / YFP
% H2A.X / YFP
SASP
Ctrl
0
2.5
25
250 nM
b
0 nM
2.5 nM
25 nM
250 nM
p21YFP
c
% p21 / YFP-
ns
ns
ns
0
2.5
25
250
nM

**Supplementary Figure 12. Use of TGFβ Inhibitor *in vitro* decreases paracrine senescence. Controls**

**(a)** Quantification of different senescent markers and BrdU incorporation in CM-SASP-treated and Control-treated YFP-Biliary cells with different concentrations of LY-2157299. No analysis is performed in this set of samples. **(b)** Decrease of p21-positive (yellow) senescent YFP-Biliary cells (green) with increasing concentrations of LY-2157299. Below, digital magnification of representative areas. **(c)** Total percentage of p21 in YFP-negative CM-SASP treated biliary cells (representative of the initial senescent biliary cells) show no variation with increasing concentrations of LY-2157299. ANOVA, Sidak's multiple comparisons test. N=4 biological replicates.

Scale bars = 50μm

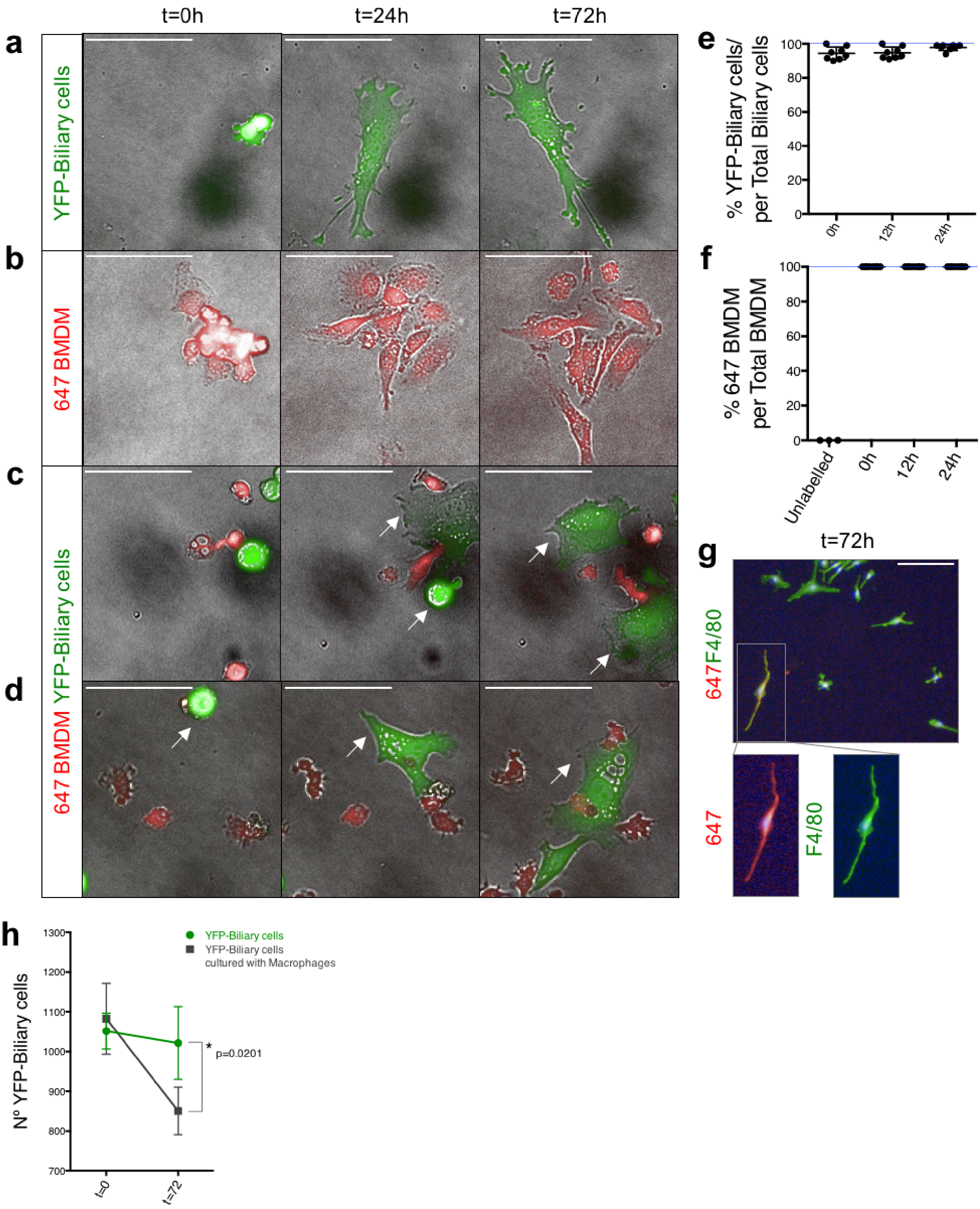

t=0h
t=24h
t=72h
a
YFP-Biliary cells
b
647 BMDM
c
d
647 BMDM YFP-Biliary cells
e
% YFP-Biliary cells/ per Total Biliary cells
100
80
60
40
20
0
0h
12h
24h
f
% 647 BMDM per Total BMDM
Unlabelled
g
t=72h
647F4/80
647
F4/80
h
N° YFP-Biliary cells
1300
1200
1100
1000
900
800
700
t=0
t=72
YFP-Biliary cells
YFP-Biliary cells cultured with Macrophages
* p=0.0201

**Supplementary Figure 13. Co-culture of senescent cholangiocytes and BMDM**

Representative images of the co-cultures of YFP-Biliary cells and 647-BMDM at 0, 24 and 72 hours. T=0 represents the cultures right after plating the different populations in ultra-low attachment plates. Images from top to bottom show:

**(a)** YFP-Biliary cells single culture (green). **(b)** 647-artificially stained BMDM single culture (red). **(c, d)** Co-culture of 647-BMDM (red) and YFP-Biliary cells (green); **c** and **d** represent two different areas of the co-culture. **(e)** Percentage of YFP in the single population of biliary cells at 0, 24 and 72h show that YFP-Biliary cells do not randomly loose YFP expression N=8 biological replicates. **(f)** Percentage of 647-BMDM from total BMDM show that BDMD do not loss 647 expression. Quantification shows 647-BMDM before staining with Cell Mask Deep Red (unlabeled), t=0, 24 and 72 hours of BMDM single culture. N= 3 biological replicates for the unlabeled; for the rest: 8 biological replicates. **(g)** 647-BMDM (red) express F4/80 (green), marker of macrophages at 72 hours. **(h)** Total number of YFP-Biliary cells cultured with 647-BMDM significantly decreases in comparison with the number of YFP-Biliary cells in single culture at 72 hours. * denotes $p < 0.05$, (Mean ± SEM). Mann-Whitney test. N=4 biological replicates.

Scale bars = 50µm.

**Supplementary Figure 14. Uncropped blots**

**Uncropped blot of Figure 3.h. WB bActin,** mouse anti-β actin (Sigma) 1 hour RT (original membrane).

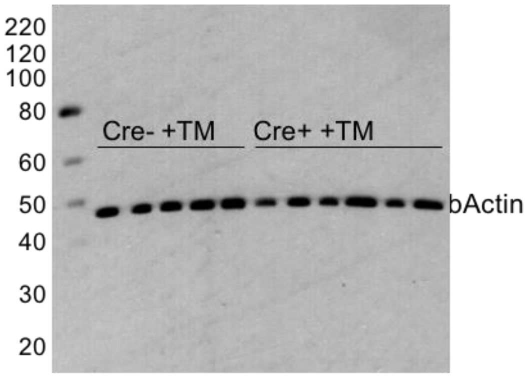


**Uncropped blot of Figure 3.h. WB NFkb,** rabbit anti-NF-kB p65 (Cell signaling). 1 hour RT (stripped membrane).

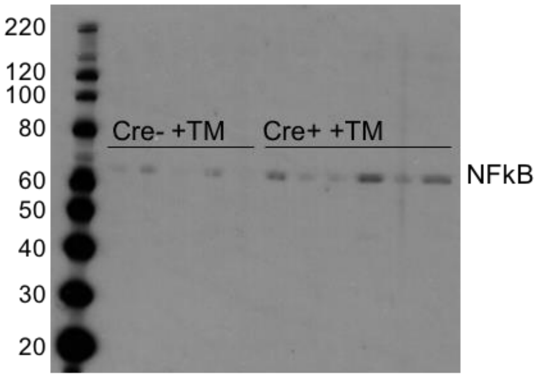


**Uncropped blot of Figure 3.h. WB pSmad2,3,** rabbit anti-p-Smad2 (Ser465/567)/Smad3 (Ser 423/4225) (Cell signaling) ON 4C. (stripped membrane).

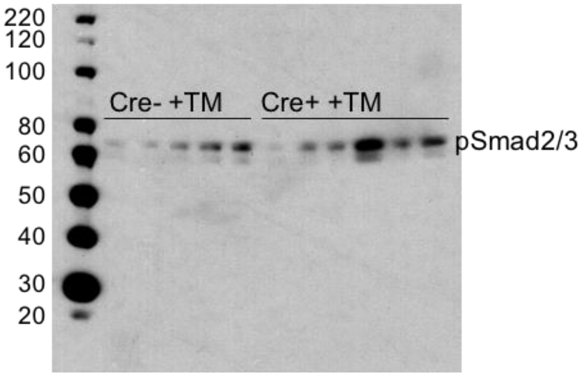

## SUPPLEMENTARY TABLES

### Supplementary Table 1. Developmental stages of PSC and PBC explants

| Classification | Ludwig | | Modified Ishak | |
|---|---|---|---|---|
| **PSC** | --- | --- | Stage 5 | *1/7* |
| | --- | --- | Stage 6 | *6/7* |
| **PBC** | Stage 4 | *7/7* | Stage 7 | *7/7* |

**Ludwig Classification** Stage 3: Biliary cirrhosis

**Modified Ishak Classification** Stage 5: Septal fibrosis, bridging necrosis or both

Stage 6: Biliary cirrhosis

### Supplementary Table 2. Primers for genotyping

| | Name | Probe Forward (F)/Reverse (R) | Internal Reporter |
|---|---|---|---|
| *Cre* | **K19CreER$^{T}$** | **F** TTAATCCATATTGGCAGAACGAAAACG<br>**R** CAGGCTAAGTGCCTTCTCTACA | CCTGCGGTGCTAACC |
| | **OPNCreER$^{T2}$** | **F** TCCTGGGCATTGCCTACAAC<br>**R** CTTCACTCTGATTCTGGCAATTTCG | ACCCTGCTGCGCATTG |
| *Reporter* | **R26RtdTomato$^{LSL}$** | **F** AGATCCACCAGGCCCTGAA<br>**R** GTCTTGAACTCCACCAGTAGTG | CCGCCGTCCTTCAGC |
| | **R26R$^{YFP}$** | **F** CACCCTCGTGACCACCTT<br>**R** GGTAGCGGGCGAAGCA | CTGCAGGCCGTAGCCG |
| | **Rosa26$^{WT}$** | **F** TTCCCTCGTGATCTGCAACTC<br>**R** CTTTAAGCCTGCCCAGAAGACT | CCGCCCATCTTCTAGAAAG |
| *Mdm2* | **Mdm2$^{Fl}$** | **F** CGAAGTTATTAGGTCTGAAGAGGAGTTT<br>**R** GCCACACCCATAATCATGTACAAGA | ACGTCCAGCCAAGCTT |
| | **Mdm2$^{WT}$** | **F** ACCCTTGCGTTTAGAAAGTACTGT<br>**R** TTTAATTTTTTAAGAGAGAATGAGATGAGTCAAAGC | CTAGTTAGCTGCCCCAGCCAG |

## Supplementary Table 3. Antibodies

| Antibody | Company | Cat. N. | Species | Reactivity | Antigen Retrieval | Dilution |
|---|---|---|---|---|---|---|
| **Primary Antibodies** | | | | | | |
| p16 Ink4a | Abcam | ab54210 | m mAb | H | C 15 min HP | 1/500 |
| 53BP1 | Abcam | ab172580 | Rb pAb | M H | C 15 min HP | 1/250 |
| DCR2 | Abcam | ab108421 | Rb pAb | M H | C 15 min HP | 1/250 |
| F4/80 | Abcam | ab6640 | R mAb | M | PK 5 min 37ºC | 1/50 |
| H2A.X | Abcam | ab81299 | Rb mAb | M H | C 15 min HP | 1/250 |
| HNF4α | Abcam | ab181604 | Rb mAb | M | C 15 min HP | 1/200 |
| Keratin-19 | DSHB | TromaIII | R mAb | M, H | C 15 min HP | 1/200 |
| Ki67 | Abcam | ab16667 | Rb mAb | M, H | C 15 min HP | 1/200 |
| p21 WAF1/Cip1 | Dako | SX118 | M mAb | H | T 10 min HP | 1/20 |
| p21 WAF1/Cip1 | Santa Cruz | SC-471 | Rb pAb | M | C 15 min HP | 1/250 |
| p27 Kip1 | Cell Signaling | 3686S | Rb mAb | M H | C 15 min HP | 1/250 |
| p53 | Abcam | ab26 | M mAb | M | C 15 min HP | 1/200 |
| p16 Ink4a | Abcam | ab108349 | Rb mAb | M | C 15 min HP | 1/100 |
| Wilms Tumor 1 | Abcam | ab89901 | Rb mAb | M | T 10 min HP | 1/300 |
| RFP | Abcam | ab62341 | Rb pAb | M | C 15 min HP | 1/200 |
| GFP | Abcam | ab6673 | G pAb | M | C 15 min HP | 1/200 |
| αSma | Sigma Aldrich | A2547 | M mAb | M | C 15 min HP | 1/2000 |
| BrdU | Abcam | ab6326 | R mAb | M | 2N HCl 30 min RT | 1/100 |
| **Secondary Antibodies** | | | | | | |
| Biotinilated Anti-Rabbit IgG (H+L) | Vector | BA-1000 | G | Rb | --- | 1/500 |
| Biotinilated Anti-Mouse IgG (H+L) | Vector | BA-9200 | G | M | --- | 1/500 |
| Anti-Rat IgG (H+L)-488 | Invitrogen | A21208 | Dk | R | --- | 1/250 |
| Anti-Rat IgG (H+L)-555 | Invitrogen | A21434 | G | R | --- | 1/250 |
| Anti-Rabbit IgG (H+L)-488 | Invitrogen | A21206 | Dk | Rb | --- | 1/250 |
| Anti-Rabbit IgG (H+L)-555 | Invitrogen | A31572 | Dk | Rb | --- | 1/250 |
| Anti-Mouse IgG (H+L)-488 | Invitrogen | A21202 | Dk | M | --- | 1/250 |
| Anti-Mouse IgG (H+L)-555 | Invitrogen | A31570 | Dk | M | --- | 1/250 |
| Anti-Mouse IgG (H+L)-647 | Invitrogen | A31571 | Dk | M | --- | 1/250 |
| Anti-Goat IgG (H+L)-488 | Invitrogen | A11055 | Dk | G | --- | 1/250 |
| Anti-Goat IgG (H+L)-555 | Invitrogen | A21432 | Dk | G | --- | 1/250 |
| **Isotype Controls** | | | | | | |
| Rat IgG | Vector | I 4000 | R | Concentration depending on 1º Ab | | |
| Rabbit IgG | Vector | I 1000 | Rb | Concentration depending on 1º Ab | | |
| Mouse IgG | Vector | I 2000 | M | Concentration depending on 1º Ab | | |
| Goat IgG | Vector | I 5000 | G | Concentration depending on 1º Ab | | |
| **Others** | | | | | | |
| DAPI for nucleic acid staining | Sigma | D9542 | | | | 1/1000 |

M, Mouse; R, Rat; Rb, Rabbit; Gt, Goat; Dk, Donkey; H, Human. mAb: monoclonal antibody; pAb: polyclonal antibody.

Antigen retrieval: C, Citrate 1x; T, Tris-EDTA 1x (HP High power); PK: Proteinase K, 20 ug/ml

**Supplementary Table 4. Primers for RT-qPCR**

| Name | Qiagen QuantiTect Primer Assay |
|---|---|
| PPIA1 | QT00247709 |
| TGFb1 | QT00145250 |
| TGFb2 | QT00106806 |
| TGFb3 | QT00166838 |
| TGFbR-I | QT00135828 |
| TGFbR-II | QT00135646 |
| IL-1A | QT00113505 |
| IL-6 | QT00098875 |
| Inh a | QT00246953 |
| Inh b | QT00245259 |
| NFkb | QT00154091 |
| HNF4a | QT00144739 |
| cdkn1a | QT00137053 |
| cdkn1b | QT01058708 |
| cdkn2a | QT00252595 |
| K19 | QT00156667 |

# SUPPLEMENTARY NOTE 1.

## ARRIVE Guidelines

(Kilkenny et al. PLOS Biology, 2010)
https://www.nc3rs.org.uk/sites/default/files/documents/Guidelines/NC3Rs%20ARRIVE%20Guidelines%202013.pdf

| ITEM | | RECOMMENDATION | |
|---|---|---|---|
| 1 | Title | Provide as accurate and concise a description of the content of the article as possible | X |
| Paracrine cellular senescence exacerbates biliary injury and impairs regeneration | | | |
| 2 | Abstract | Provide an accurate summary of the background, research objectives, including details of the species or strain of animal used, key methods, principal findings and conclusions of the study | X |
| Cellular senescence is a mechanism that provides an irreversible barrier to cell cycle progression to prevent undesired proliferation. However, under pathological circumstances, senescence can adversely affect organ function, viability and regeneration.<br>We have developed an efficient mouse model of biliary senescence, based on the conditional deletion of Mdm2 in bile ducts under the control of the Krt19 promoter that exhibits features of biliary disease. We used this model to study the effects of cholangiocyte senescence in biliary injury and how this mechanism affects the regenerative capacity of the parenchyma.<br>Senescent cholangiocytes induce profound alterations in the cellular and signaling microenvironment with recruitment of myofibroblasts and macrophages causing collagen deposition, TGFβ production and induction of senescence in surrounding cholangiocytes and hepatocytes. Finally, we studied how inhibition of TGFβ-signalling disrupts the transmission of senescence and restores liver function.<br>We have identified cellular senescence as a detrimental mechanism in the development of biliary injury. Our results identify TGFβ as a potential therapeutic target to limit senescence-dependent aggravation in human cholangiopathies. | | | |
| INTRODUCTION | | | |
| 3 | Background | a. Include sufficient scientific background (including relevant references to previous work) to understand the motivation and context for the study, and explain the experimental approach and rationale.<br>b. Explain how and why the animal species and model being used can address the scientific objectives and, where appropriate, the study's relevance to human biology. | X<br>X |
| **a.** Primary Sclerosing Cholangitis (PSC) and Primary Biliary Cholangitis (PBC) are the most prevalent type of cholangiopathies, a diverse group of genetic and acquired disorders that affect the biliary population of the liver[1-3]. PSC/PBC have variable prognoses but frequently evolve into end-stage liver disease, with limited treatment options.<br>The aetiologies remain unclear, although a role of cellular senescence in the development of PSC/PBC has been suggested[4-8]. Senescence is an irreversible cell cycle arrest, driven by dominant cell-cycle inhibitors and characterized by changes in morphology, increased lysosomal content[9], expression of DNA damage response (DDR) factors[10], and the activation of the senescence-associated secretory phenotype (SASP)[11, 12]. The SASP is a pro-inflammatory response that activates and reinforces the senescent phenotype in the surrounding cells[12], modulates fibrosis[13] and promotes regeneration[11, 14].<br>However, despite a number of studies suggesting a potential link between senescence and biliary disease[4-8], it has not been shown whether senescence is actually a driver of the damage rather than solely a consequence[8]. We have therefore investigated the relationship between senescence and biliary disease, focusing on SASP-related mechanisms to explain part of the pathophysiology of PSC/PBC. In doing so we provide potential opportunities for novel therapies based on the disruption of the TGFβ-dependent SASP response.<br><br>**References in this section include seminal papers on the field such as:** Lazaridis, Strazzabosco and Larusso 2004; Hirschfield et al., 2013; Harada et al., 2001; Sasaki et al., 2010; Tabibian et al., 2014. | | | |
| **b.** Previous studies have described the presence of senescent cholangiocytes in human sections of PSC and PBC, and conferred to this phenomenon potential causality in the development of biliary disease (see Harada et al., 2001; Sasaki et al., 2010; Tabibian et al., 2014). This seminal papers provide the proof of concept of the deleterious implications of cellular senescence in the context of PSC and PBC.<br>However, to completely understand the dynamics of cellular senescence in biliary disease and to fully address its importance, *in vivo* models are necessary. Our K19-Mdm2 model specifically induces p21-dependent cell cycle arrest in cholangiocytes, allowing the study of the effects of cellular senescence in the development of biliary disease. Furthermore, experiments in this K19-Mdm2 line highlighted the importance of TGFb signaling in the expansion of paracrine senescence, providing potential opportunities of novel therapies based on the disruption of TGFb. | | | |
| 4 | Objectives | Clearly describe the primary and any secondary objectives of the study, or specific hypotheses being tested | X |
| 1. To generate a murine model that conditionally displays cellular senescence in cholangiocytes. By using this model:<br>- To determine the effects of cellular senescence in the development of biliary injury. | | | |

- To study the effects of the expansion of cellular senescence (SASP-mediated) into the parenchyma.
- To determine which factors of the SASP are specifically involved in this settings.
- To target those factors and assess if a partial interruption of the SASP restores the phenotype of the model.

## METHODS

| | | | |
|---|---|---|---|
| 5 | **Ethical Statement** | Indicate the nature of the ethical review permissions, relevant licenses (e.g. Animal [Scientific Procedures] Act 1986), and national or institutional guidelines for the care and use of animals, that cover the research. | X |

All animal experiments were carried our under procedural guidelines, severity protocols and within the UK with ethical permission from the Animal Welfare and Ethical Review Body (AWERB) and the Home Office (UK).

| | | | |
|---|---|---|---|
| 6 | **Study design** | For each experiment, give brief details of the study design including:<br>a. The number of experimental and control groups<br>b. Any steps taken to minimise the effects of subjective bias when allocating animals to treatment (e.g. randomisation procedure) and when assessing results (e.g. if done, describe who was blinded and when).<br>c. The experimental unit (e.g. a single animal, group or cage of animals)<br>A time-line diagram or flow chart can be useful to illustrate how complex study designs were carried out. | X |

**1. Induction and characterization of the model. Figure 2 and Supplementary Figures 2, 3, 4, 5, 6 and 7**. This experiment aims to characterise the K19-Mdm2 model after tamoxifen induction. For that purpose, we administered tamoxifen and assessed different markers of senescence on different days (Cre+ +TM, Day 2, Day 21, Day 60 and Day 90). We included Cre-negative K19-Mdm2 controls (Cre-+TM) to assess the specificity of the tamoxifen dependent- induction and healthy controls to assess the basal levels of senescence. We also compared K19-Mdm2 response with the OPN-Mdm2 model, a similar murine model that harbours a different promoter to target cholangiocytes.
**a.** Healthy untreated (WT): N=6; Cre- +TM Day 2: N=6; Cre+ +TM Day 2: n=6; Cre+ +TM Day 21: N=6; Cre- +TM Day 21: N=4; Cre+ +TM Day 60: N=6; Cre+ +TM Day 90: N=4). OPN-Mdm2 Healthy untreated: N=6; OPN-Mdm2 Cre+ +TM Day 2 N=6.
**b.** Mice were randomly assigned to each group. Histological sections were assigned a blinded code prior quantification and the randomization decoded at the end of the final data analysis.
**c.** Mice were grouped in cages according to sex and treatment for the duration of the experiment.

**2. Assessing SASP's factors in bile ducts and hepatocytes. Figure 3 and Supplementary Figures 8 and 9**. This experiment aims to identify SASP factors in the transmission of senescence (from the induced cholangiocytes towards hepatocytes). For that purpose, we isolated bile ducts and hepatocytes at day 2 after tamoxifen administration in Cre- vs Cre+.
**a.** For bile duct isolation (Cre- +TM: N=5; Cre+ +TM: N=6). For Hepatocyte isolation (Cre- +TM: N=5; Cre+ +TM N=6).
**b.** Mice were randomly assigned to each group.
**c.** Mice were grouped in cages according to sex and treatment for the duration of the experiment.

**3. Exploring the role of senescence during dietary-induced biliary damage. Figure 4 and Supplementary Figure 10**. This experiment aims to study the role of senescent cholangiocytes in the K19-Mdm2 model during a secondary biliary insult such as DDC diet, which has been previously described as a PSC model (Fickert et al., 2007). This experiments allowed us to disclose the detrimental effects of senescence in the bile ducts and hepatic parenchyma. We included a group of uninduced K19-Mdm2 mice in which we administered DDC diet (DDC), to assess the effects of the diet itself. Experimental group include K19-MDM2 mice in which we administered tamoxifen, waited two days until the display of senescence, and then administered DDC diet (S+DDC).
**a.** DDC: N=6; S+DDC: N=8.
**b.** Mice were randomly assigned to each group.
**c.** Mice were grouped in cages according to sex and treatment for the duration of the experiment.

**4. Exploring the effects of cholangiocyte-derived paracrine senescence into the regenerative potential of liver parenchyma. Figure 5 and Supplementary Figure 11**. In this experiment we compared the regenerative potential of 70% Partial Hepatectomy 2 days after inducing senescence in the model (S+PH). As controls, we included a group that only received the Hepatectomy (PH). We also included a Sham control to assess the specific effects of the Hepatectomy (Sham).
We observed two time points (48 hours and 7 days after the surgery).
**a.** At 48 hours: S+PH: N=7; PH: N=8; Sham: N=4.
At 7 days: S+PH: N=6; PH: N=4.
**b.** Mice were randomly assigned to each group.
**c.** Mice were grouped in heated cages in pairs after the surgery (maintaining same sex and treatment).

**5. Assess the effects of partial ablation of paracrine senescence effects. Figure 7**. In this experiment we analysed the effects of different doses of LY2157299 (TGFb inhibitor, administered by oral gavage) and the effects of LY2157299 in the context of senescence and biliary injury (S+DDC).
**a.** To assess drug concentration: Vehicle: N=5; 10 mg/ml: N=5; 20 mg/ml: N=5.
To assess the effects of ablation of paracrine senescence during biliary injury: Vehicle: N=5; Inhibitor: N=5.
**b.** Mice were randomly assigned to each group.
**c.** Mice were grouped in heated cages in pairs after the surgery (maintaining same sex and treatment).

| | | | |
|---|---|---|---|
| 7 | **Experimental procedures** | For each experiment, and each experimental group, including controls, provide precise details of all procedures carried out. For example:<br>a. How (e.g. drug formulation and dose, site and route of administration, anaesthesia and analgesia used [including monitoring], surgical procedure, method of euthanasia. Provide details of any specialist equipment used, including suppliers(s).<br>b. When (e.g. time of the day)<br>c. Where (e.g. home cage, laboratory, water maze).<br>d. Why (e.g. rationale for choice of specific anaesthetic, route of administration, drug dose used). | X |

**For all the experiments:**
Euthanasia for tissue collection was performed by schedule 1 approved method (dislocation of the neck).

**1. Induction of the K19-Mdm2 and OPN-Mdm2 models:**
**a, b.** Three doses of tamoxifen/vehicle are administered to the mice by intraperitoneal route, every other day (in a Monday-Wednesday-Friday pattern). Injections were performed between 9-10 am with sterile insulin syringes. Tamoxifen (4 mg, Sigma) is dissolved in sunflower seed oil (Sigma) and stored at -20ºC until use, where it is defrosted, sonicated and maintained at room temperature prior injection. 200 ul are administered per mice. Control mice are administered with the same quantity of vehicle (200 ul of sunflower seed oil) in the same pattern.
**c.** Tamoxifen/oil was administered in the animal unit, immediately placing the mice in their cages.
**d.** This pattern of administration is the result of previous experiments in the lab where we optimised the route of administration for the optimal recombination rates of similar models. However, further experiments with repetitive oral gavages of low doses of tamoxifen proved to be equally efficient.

**2. For the isolation of Bile ducts and Hepatocytes in the induced K19-Mdm2 model.**
**a. b, c, d.** A similar pattern as for section 1 (see above) was followed. Mice were induced and caged for two days until the display of senescent cholangiocytes. At that point, bile ducts and hepatocytes were isolated following standard techniques (see Supplementary Methods).

**3. DDC-induced biliary injury.**
**a, b.** Mice were induced as previously described (see above, section 1). 0.1% 3,5-Diethozycarbonyl-1,4-dihydrocollidine (DDC) mixed with Rat & Mouse No Maintenance (RM1) diet (mix provided by Special Diet Services) is administered to the mice two days after the last administration of tamoxifen, and maintained for 1 week. Mice were weighted every day to assess weight loss.
**c.** DDC diet was placed on the cage so the mice had free access to the food.
**d.** Although the literature describes prolonged periods of DDC feeding in different mouse models, the K19-Mdm2 line only admitted one week of DDC. This decision takes into consideration restrictions of a maximum total weight loss of 20%.

**4. Partial Hepatectomy.**
**a.** Prior surgery, and under anaesthesia, animals were administered 100 ul of analgesia (Rymadil) subcutaneously.
Briefly, median laparotomy was performed under inhaled isoflurane anaesthesia and left lateral and median lobe were removed as previously described (Mitchell and Willenbring, 2008). To avoid damaging the gallbladder, the 'three knots' technique was performed. Sham-operated mice were subject to laparotomy and brief manipulation of the intestines, but no Hepatectomy was performed. Mice were sacrificed at 48 hours (peak of hepatocyte DNA replicative rate) and 1 week (usual recovery of the resected liver mass) after surgery.
After surgery animals were kept in heated cage (25ºC), mash was placed in the floor of the cage to allow a better access to the food and mice were carefully monitored every 12 hours for any signs of distress. Two more doses of analgesia were administered at 12 and 24 hours post-surgery.
**b.** Partial Hepatectomy was performed between 6 and 8 o clock in the morning to avoid side effects of circadian rhythms in the proliferative potential of the parenchyma. Consequently, the number of mice that were used for surgery per day were limited to a maximum of 4 (from the beginning to the end, it takes us approximately 20-30 minutes to perform the entire surgery).
**c.** Surgery was performed in a sterile hood, with the animals placed on a heat pad at 28ºC degrees. The are of incision is covered with a sterile surgical pad. Sterile gloves, sterile vycril sutures and sterile tools were used during all the procedure. Gloves were changed between mice and surgical tools were sterilised using a bead-steriliser. Warm sterile saline was administered to the cavity to avoid dehydration of the tissues.
d. We chose isoflurane compared with the usual intraperitoneal Ket/Met dose as the animals recover faster and better from the surgery. By keeping the animals in a heat pad after the surgery, administering analgesia and facilitating the access to food we minimise the post-operative stress.

**5. Use of LY2157299.**
**a.** Galunisertib (LY2157299, Cayman Chemical Company) was administered at 10 mg/kg twice daily by gavage after the induction of the model until day 2, when DDC diet was administered for 1 week. LY2157299 was prepared in 10% polyethylene glycol, 5% DMSO and 85% saline vehicle. Control mice were administered only with the vehicle (PEG, DMSO, Saline).
**b.** Mice were administered at 9 am and 7 pm.

**c.** LY2157299 was administered in the animal unit, immediately placing the mice in their cages.
**d.** We chose preferentially this drug as it has shown less cardiac toxicity in comparison with other TGFb inhibitors (Herbertz et al., 2015), while efficiently inhibiting TGFb signalling pathway.

| | | | |
|---|---|---|---|
| 8 | **Experimental animals** | a. Provide details of the animals used, including species, strain, sex, developmental stage (e.g. mean or median age plus age range) and weight (e.g. mean or median weight plus weight range).<br>b. Provide further relevant information such as the source of animals, international strain nomenclature, genetic modification status (e.g. knock-out or transgenic), genotype, health/immune status, drug or test naïve, previous procedures, etc. | X |

**The K19Mdm2 model:**
**a.** K19CreER$^{T}$+ and K19CreER$^{T}$- mice were crossed with Mdm2$^{flox/flox}$ and R26RtdTomato$^{LSL}$ mice to generate K19-Mdm2$^{flox/flox}$tdTom line (Cre+ and Cre-). The animals used on this study are C57Bl/6 background. Mix of males and females between 8-12 weeks old were used for the experiments (20-25 g).
**b.** K19CreER$^{T}$ were purchased from Jackson Laboratory. Mdm2$^{flox/flox}$ mice were generously provided by Professor Gigi Lozano, Department of Cancer Genetics, University of Texas. R26RtdTomato$^{LSL}$ were purchased from Jackson Laboratory.

**The OPNMdm2 model:**
**a.** OPNiCreER$^{T2}$+ and OPNiCreER$^{T2}$- mice were crossed with Mdm2$^{flox/flox}$ and R26R$^{YFP}$ mice to generate OPN-Mdm2$^{flox/flox}$YFP line (Cre+ and Cre-). OPN-Mdm2$^{flox/flox}$YFP line have a mixed (C57BL6/J and CD1) background. Mix of males and females between 8-12 weeks old were used for the experiments (20-25 g).
**b.** OPNiCreER$^{T2}$R26R$^{YFP}$ mouse line was a kind gift by F. Lemaigre and I. Leclercq, Institut de Recherche Expérimentale et Clinique and Duve Institute Brussels, Belgium.

| | | | |
|---|---|---|---|
| 9 | **Housing and husbandry** | Provide details of:<br>a. Housing (type of facility e.g. specific pathogen free [SPF]; type of cage or housing; bedding material; number of cage companions, tank shape and material, etc, for fish).<br>b. Husbandry condition (e.g. breeding programme, light/dark cycle, temperature, quality of water etc for fish, type of food, access to food and water, environmental enrichment).<br>c. Welfare-related assessments and interventions that were carried out prior to, during or after the experiment. | X |

**a.** Mice were housed in a specific pathogen free (SPF) environment in open cages (NKP, M3- sloping front). Overall size of the cage is 48x15x13 cm. Internal size 510 cm$^{2}$x13 cm. Bedding is Aspen chips. A minimum of two mice are kept per cage (maintaining in the same cage animals of same sex, and same treatment when required). A maximum of 6 mice are kept per M3 cage.
**b.** Light cycle is 14 hours (On at 5 am, Off at 19 pm), with a temperature of 21ºC. Access to food (Irradiated RM3P) and water *ad libitum*.
**c.** Daily check for sick/dead mice are performed. Health screening of the facility are performed every 3 months.
During special diets (such as DDC) and after surgical procedures (partial hepatectomy) daily weights are taken to assess the welfare of the animal. If weight loss exceeds 20%, mice are closely monitored and should the animal not gain weight, they are culled by cervical dislocation and not included in further analysis. Should the weight loss exceed 30% mice are culled by cervical dislocation and are not included in further analysis.

| | | | |
|---|---|---|---|
| 10 | **Sample size** | a. Specify the total number of animals used in each experiment, and the number of animals in each experimental group.<br>b. Explain how the number of animals was arrived at. Provide details of any sample size calculation used.<br>c. Indicate the number of independent replications of each experiment, if relevant. | X |

**a. Total number of animals, and number of animals per experimental group**

**1. Induction and characterization of the model. Figure 2 and Supplementary Figures 2, 3, 4, 5, 6 and 7**.
Total N=49.
K19-Mdm2: Healthy (N=6); Cre- +TM day 2 (N=6); Cre+ +TM day 2(N=6); Cre+ +TM day 21 (N=6); Cre+ +TM day 60 (N=5); Cre+ +TM day 90 (N=4).
OPN-Mdm2: Healthy (N=6); Cre+ +TM day 2 (N=6).
**2. Assessing SASP's factors in bile ducts and hepatocytes. Figure 3 and Supplementary Figures 8 and 9**.
Total N=22
K19-Mdm2: Cre-+TM (N=5 for bile duct isolation, N=5 for hepatocyte isolation)
Cre+ +TM (N=5 for bile duct isolation, N=5 for hepatocyte isolation)
**3. Exploring the role of senescence during dietary-induced biliary damage. Figure 4 and Supplementary Figure 10.**
Total N=14
K19-Mdm2: Senescence + DDC (S+DDC) N=8; DDC Control (DDC) N=6.
**4. Exploring the effects of cholangiocyte-derived paracrine senescence into the regenerative potential of liver parenchyma. Figure 5 and Supplementary Figure 11**.
Total N=29
K19-Mdm2: 48hours post-PH: Sham N=4; 70% PH N=8; S+PH N=7.

K19-Mdm2: 1 week post-PH: PH N=4; S+PH N=6.
**5. Assess the effects of partial ablation of paracrine senescence effects. Figure 7**.
Total N=25
K19-Mdm2: Vehicle N=5; 10 mg/ml LY2157299 N=5; 20 mg/ml LY2157299 N=5.
K19-Mdm2: Vehicle+S+DDC N=5; Inhibitor LY2157299+S+DDC N=5.

**b. Power calculations**
Power calculations were not routinely performed. However, animal numbers were chosen to reflect the expected magnitude of response taking into account the variability observed in previous experiments.

| | | | |
|---|---|---|---|
| **11** | **Allocating animals to experimental groups** | a. Give full details on how animals were allocated to experimental groups, including randomisation or matching if done.<br>b. Describe the order in which the animals in the different experimental groups were treated and assessed. | X |

**a.** Mice were randomly allocated to each experimental group. Males and females were equally distributed among the groups. Each mouse was assigned a number for the rest of the experiment.
**b.** Control groups (in which oil was administered) were treated before experimental groups (in which tamoxifen was administered) to avoid cross-contamination.
All groups receive the same diet (or control diet) at the same time.
For surgical procedures, a mix of control and experimental mice were included per surgery.

| | | | |
|---|---|---|---|
| **12** | **Experimental outcomes** | Clearly define the primary and secondary experimental outcomes assessed (e.g. cell death, molecular markers, behavioural changes). | X |

Refer to the results section

| | | | |
|---|---|---|---|
| **13** | **Statistical methods** | a. Provide details of the statistical methods used for each analysis<br>b. Specify the unit of analysis for each dataset (e.g. single animal, group of animals, single neuron).<br>c. Describe any methods used to assess whether the data met the assumptions of the statistical approach. | X |

For specific data, refer to the results section.
**a.** A minimum of 4 mice were included per experimental group. For cell culture experiments, a minimum of four independent biological replicates were performed.
Prism software version 5.0a (GraphPad Software, Inc) was used for all statistical analysis. Normal distribution of data was determined using D'Agostino and Pearson omnibus normality test with Welch's correction if variances differed (F test). For parametric data, data significance was analysed using a two-tailed unpaired Students t-Test. Non-parametric data was analysed using Mann-Whitney test. In cases where more than two groups were being compared, then a one-way ANOVA (with Bonferroni correction) was used. Survival curves are calculated using Log-Rank comparison (Mantel-Cox test) and Gehan-Breslow-Wilcoxon test.
Statistical significance was assumed at $p<0.05$. Data is presented as mean ± standard error of the mean (SEM). N refers to biological replicates.

## RESULTS

| | | | |
|---|---|---|---|
| **14** | **Baseline data** | For each experimental group, report relevant characteristics and health status of animals (e.g. weight, microbiological status, and drug or test naïve) prior to treatment or testing (this information can often be tabulated). | X |

For specific data, refer to the results section.
All mice were between 8-12 weeks (20-25 g of weight at the beginning of the experiment) in SPF environment.

| | | | |
|---|---|---|---|
| **15** | **Numbers analysed** | a. Report the number of animals in each group included in each analysis. Report absolute numbers (e.g. 10/20, not 50%)<br>b. If any animals or data were not included in the analysis, explain why. | X |

For specific data, refer to the results section.
**a.** Data are represented as scatter dot-plot, therefore each animal is represented by one dot. For the survival curve, **Figure 5** absolute numbers are included alongside the graph.
**b.** All mice used for each experiment were included in the statistical analysis.

| | | | |
|---|---|---|---|
| **16** | **Outcomes and estimation** | Report the results for each analysis carried out, with a measure of precision (e.g. standard error or confidence interval) | X |

For specific data, refer to the results section.
Statistical significance was assumed at $p<0.05$. Data is presented as mean ± standard error of the mean (SEM).

| | | | |
|---|---|---|---|
| **17** | **Numbers analysed** | a. Give details of all important adverse events in each experimental group.<br>b. Describe any modifications to the experimental protocols made to reduce adverse events. | X |

**a.** In **Figure 7**, administration of 20 mg/ml of LY2157299 lead to a greater decrease of senescence levels when compared with the 10 mg/ml group. However, 20 mg/ml dose also lead to increased toxicity assessed by ALT/AST levels in serum (data not shown). Therefore, we decided to perform subsequent experiments with the 10 mg/ml dose of LY2157299.
In **Figure 4**, administration of DDC diet lead to a quick loss of weight.
**b.** Although the literature describes the use of DDC diet for prolonged periods of time, to minimise weight loss, preserve the welfare of the mice, and avoid off-target effects, we restricted DDC diet administration to 1 week.

## DISCUSSION

| | | | |
|---|---|---|---|
| **18** | **Interpretation/ scientific implications** | a. Interpret the results, taking into account the study objectives and hypotheses, current theory and other relevant studies in the literature<br>b. Comment on the study limitations, including any potential sources of bias, any limitations of the animal mode, and the imprecision associated with the results.<br>c. Describe any implications of your experimental methods or findings for the replacement, refinement or reduction (the 3Rs) of the use of animals in research. | X |

**a.** Refer to the Discussion section.
**b.** In our paper, we disclose the positive effect of TGFb inhibition to avoid the expansion of senescence. Among other positive effects, fibrosis was also decreased after the use of TGFb inhibitors. However, it it should be noted that TGFb inhibition can also decrease liver fibrosis *per se* (Dooely and ten Dijke, 2012).
**c.** NA.

| | | | |
|---|---|---|---|
| **19** | **Generalisability/ translation** | Comment on whether, and how, the findings of this study are likely to translate to other species or systems, including any relevance to human biology. | X |

Overall, we have shown that cellular senescence is likely to be a driver of biliary injury by affecting the microenvironment, impairing liver parenchyma regeneration and impairing biliary function and found this pathology can be specifically targeted through TGFβ inhibition. This could be potentially applied in the future to clinical therapies for biliary disease.

| | | | |
|---|---|---|---|
| **20** | **Funding** | List all funding sources (including grant number) and the role of the funder(s) in the study | X |

S.F.G. was funded by Principal's Career Development PhD Scholarships, Edinburgh University, UK
This work was supported by the UK Medical Research Council.
